# Poverty-modulated tree equity shapes health burdens across U.S. cities

**DOI:** 10.1016/j.ese.2026.100758

**Published:** 2026-09-03

**Authors:** Min-Ki Lee, Chang-Bae Lee, Sungyong You, Stephen J. Freedland, Michael R. Freeman

**Affiliations:** a Department of Forest Resources, Kookmin University, Seoul, 02707, Republic of Korea; b Department of Climate Convergence Technology, Kookmin University, Seoul, 02707, Republic of Korea; c Department of Urology, Cedars-Sinai Medical Center, Los Angeles, CA, 90048, USA; d Department of Computational Biomedicine, Cedars-Sinai Medical Center, Los Angeles, CA, 90048, USA; e Department of Biopharmaceutical and Regulatory Science, School of Pharmacy, Sungkyunkwan University, Suwon, 16419, Republic of Korea; f Department of Biomedical Sciences, Cedars-Sinai Medical Center, Los Angeles, CA, 90048, USA

**Keywords:** Environmental exposure, Socioeconomic inequality, Urban green space, Urban health inequities, Environmental justice

## Abstract

Rapid urbanization concentrates both opportunity and health risk across cities worldwide. Although urban green infrastructure is widely promoted for public health, most evidence examines single outcomes or simple area metrics and rarely tests how socioeconomic context modulates the magnitude and pathways of these benefits. Whether tree-canopy equity delivers stronger returns in areas with the highest poverty remains unresolved. Here, we show that poverty-modulated tree equity shapes health burdens across 497 cities in the United States. Socioeconomic disadvantage is the dominant structural driver of eleven physical and mental health outcomes, yet higher Tree Equity Score is consistently associated with lower prevalence; these protective associations are strongest in high-poverty cities, where greening acts partly by mitigating heat and air-pollution hazards, whereas in low-poverty cities direct restorative pathways predominate. Models explain up to 72.6 percent of the variation in poor mental health, and a five-point rise in the Tree Equity Score corresponds to a 2.7–35 percent lower prevalence of multiple conditions. These findings demonstrate that the health returns of urban greening are context-dependent and require equity-focused strategies stratified by city poverty level.

## Highlights

• Poverty-modulated tree equity shapes health burdens across 497 U S. cities.
• Protective associations of Tree Equity Score are strongest in high-poverty cities.
• Greening pathways shift from hazard mitigation to restoration as poverty declines.
• A five-point Tree Equity Score rise links to 2.7–35 percent lower health burdens.
• Equity-focused greening must be stratified by city poverty level to maximize gains.

## Graphical abstract

**Figure:**
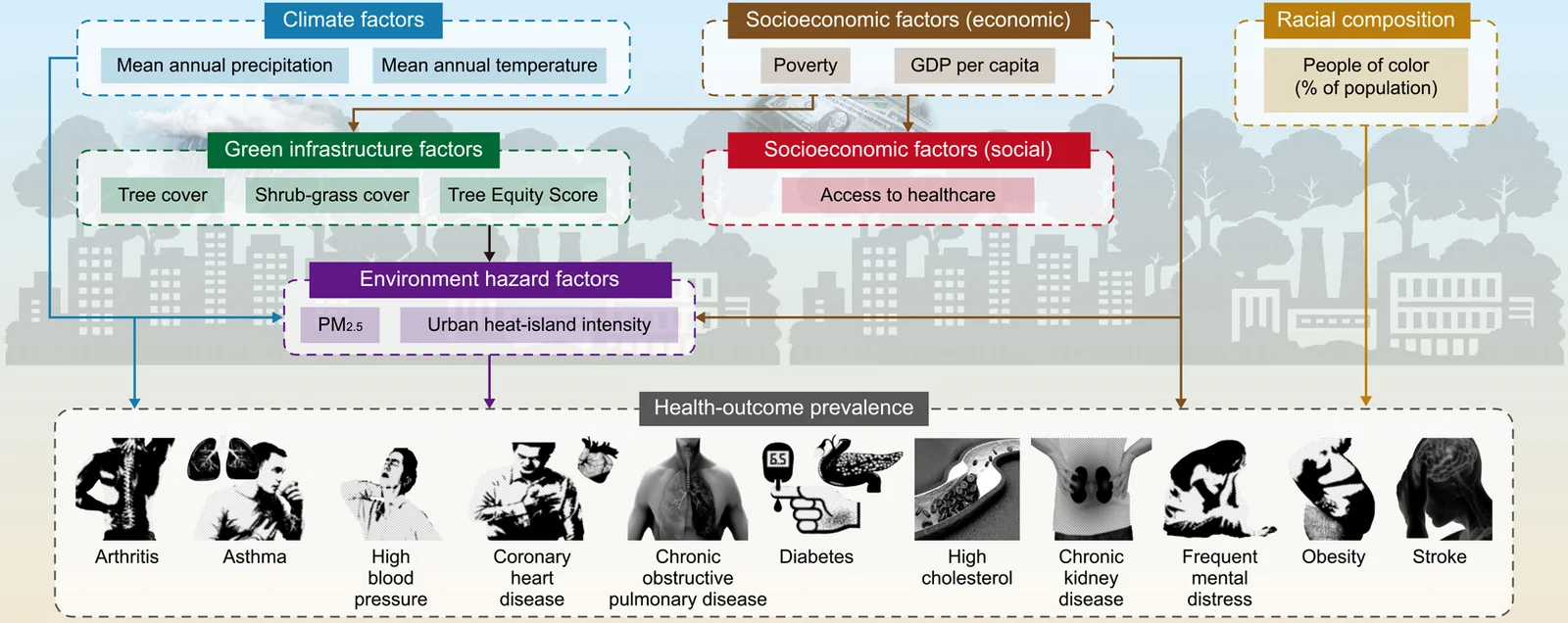

## 1 Introduction

Rapid urbanization is reshaping human living environments worldwide. The proportion of the global population residing in cities has increased from approximately 30% in 1950 to 58% in 2024 and is projected to reach 68% by 2050 [1]. Although urbanization has improved quality of life for urban residents across various domains, including transportation, housing, healthcare, and education, it has also introduced new challenges, such as environmental pollution and the rapid and widespread conversion of natural ecosystems [2]. The loss of natural vegetation reduces key ecosystem functions and services, as well as opportunities for everyday contact with nature, with potential consequences for mental and physical health by limiting pathways to psychological restoration, physical activity, and stress relief [3,4].

In this context, urban green spaces have emerged as critical components of healthy cities. They provide a range of direct and indirect health benefits and are increasingly recognized in international frameworks that address the interconnected challenges of biodiversity loss, climate change, and human health. The Kunming–Montreal Global Biodiversity Framework, adopted under the Convention on Biological Diversity, recognizes the importance of conserving and restoring urban ecosystems for human health and well-being [5]. Similarly, the World Health Organization's Urban Health Initiative calls for integrating health considerations into urban planning [6,7].

Epidemiological and ecological studies have linked greenness and urban green space with diverse health outcomes, including mental health [8,9], cardiovascular disease [10,11], respiratory disease [12,13], diabetes [4,14], obesity [15,16], and stroke [11,17]. Evidence suggests that exposure to green space can provide substantial health benefits. Geary et al. [9] reported that greater greenness and access to green spaces were associated with improved mental health in a longitudinal study of 2.3 million adults in Wales. Similarly, Yuan et al. [11] found that greater green space exposure was associated with lower mortality risk and better cardiovascular outcomes among older adults in a systematic review and meta-analysis. Proposed mechanisms include promoting physical activity, reducing psychological stress and attention fatigue, providing opportunities for social interaction, enhancing microbial diversity, and mitigating environmental hazards such as urban heat islands and fine particulate matter [[18], [19], [20]]. However, recent reviews have highlighted methodological limitations and inconsistent findings in the evidence base. Most studies focus primarily on mental health rather than on multiple chronic diseases, including cardiovascular and respiratory diseases [13,16,19,21,22]. Although human health is jointly shaped by green infrastructure, climate [23,24], socioeconomic disadvantage [25,26], racial composition [[27], [28], [29]], and environmental hazards such as heat and air pollution [24,30], few studies have compared their relative contributions or interrelationships [21,31]. The health effects of green infrastructure may vary across urban contexts characterized by income, poverty, and racial segregation, yet comparative evidence across city types and poverty levels remains limited [32,33]. Finally, research has often measured green space primarily by area or proportion, overlooking qualitative and equity-related dimensions such as accessibility, distribution, tree canopy equity, and biodiversity [9,31].

To address these gaps, we used harmonized data from the Population Level Analysis and Community Estimates (PLACES) program of the Centers for Disease Control and Prevention (CDC) [34] to examine the joint associations of environmental, socioeconomic, and demographic factors with the prevalence of 11 physical and mental health outcomes across 497 cities in the United States. We had two objectives: first, to determine how green infrastructure, climate, socioeconomic conditions, racial composition, and urban environmental hazards explain variation in disease prevalence, and second, to compare the relative importance and health-related pathways of these factors across poverty-based city groups and multidimensional city clusters. This framework is intended to clarify where equity-focused urban greening, alongside broader social and health policies, may be most effective in reducing urban disease burdens and narrowing health inequities.

## 2 Materials and methods

### 2.1 Study sites and health outcomes

We extracted health-outcome data for 500 cities from PLACES: Local Data for Better Health (hereafter, PLACES) [34]. PLACES provides model-based small-area estimates of health and health-related indicators rather than directly observed disease prevalence for areas across the United States, including counties, incorporated places, census-designated places, census tracts, and ZIP Code Tabulation Areas. These estimates combine survey data with demographic and geographic information within a small-area estimation framework. Among the health outcomes available in PLACES, we examined 11 prevalence measures: arthritis, asthma, chronic kidney disease (CKD), chronic obstructive pulmonary disease (COPD), coronary heart disease (CHD), frequent mental distress, diabetes, high blood pressure, high cholesterol, obesity, and stroke.

We excluded two additional indicators—cancer prevalence and all teeth lost because they were not conceptually aligned with the environmentally mediated chronic conditions here. The cancer measure aggregates multiple cancer types with distinct etiologies, latency periods, genetic influences, behavioral risk factors, screening practices, and treatment histories [[35], [36], [37]]. Consequently, associations between overall cancer prevalence and environmental or socioeconomic conditions are difficult to interpret within a city-level cross-sectional ecological framework. For example, elevated cancer prevalence in one city may be driven primarily by cancers associated with environmental exposures, whereas in another city it may reflect cancers more strongly influenced by genetic susceptibility or screening practices. Because cancer-specific information is not available in the CDC PLACES dataset, the aggregated cancer indicator may obscure meaningful ecological relationships and increase interpretational uncertainty. Similarly, all teeth lost reflects the cumulative consequences of multiple oral diseases and long-term life-course exposures, including aging, access to dental care, and accumulated socioeconomic disadvantage, rather than the prevalence of a specific disease [38]. This indicator represents cumulative lifetime health burden and may not be readily linked to contemporary environmental conditions. Both indicators were excluded to maintain conceptual consistency across health outcomes and to facilitate more ecologically interpretable comparisons across diseases. Of the 500 cities with available health outcome data, three cities were excluded from the analysis. Macon–Bibb County, Georgia, was excluded due to its administrative consolidation beginning in 2018, and Honolulu, Hawaii, was excluded due to changes in the geographic boundaries used to report health outcomes. Anchorage, Alaska, was also excluded because several variables, including access to healthcare, differed substantially from those of the other cities, raising concerns that it may behave as an outlier in the analysis.

Consequently, the final dataset comprised prevalence estimates for 11 health outcomes across 497 cities (Supplementary Table S1 and Fig. S1). Health-outcome prevalence values were obtained from the 2019–2021 releases of the CDC PLACES dataset, which provide estimates for 2017–2019. To minimize potential distortions associated with the COVID-19 pandemic, we included only estimates from 2017 to 2019 in the analysis, excluding estimates for 2020 and 2021. For each city, prevalence values were calculated as the mean of the annual estimates for 2017 to 2019 to match the temporal coverage of the explanatory variables. Estimates for 2019 were unavailable for nine cities in New Jersey—Camden, Clifton, Elizabeth, Jersey City, Newark, Passaic, Paterson, Trenton, and Union City—so we calculated means using the available 2017 and 2018 estimates.

### 2.2 Data sources and quantification of explanatory variables

In this study, we used four green infrastructure measures: the proportions of tree cover and shrub-grass cover, representing the quantity and composition of urban green space; the normalized difference vegetation index (NDVI), reflecting vegetation condition and productivity; and the Tree Equity Score (TES), a qualitative index reflecting equity in access to urban trees. City-level area data used to calculate green space proportions were obtained from the CDC city-boundary shapefiles. Green-space areas were quantified using the Esri Sentinel-2 Land Cover Explorer, an annual Sentinel-2-based land-cover dataset with a spatial resolution of 10 m [39,40]. The dataset includes nine land-cover classes. We defined the “tree” and “shrub-grass” classes as urban green space and extracted their areas for each city. We then calculated the proportions of total green space, tree canopy cover, and shrub-grass cover relative to total city area. Annual values for 2017–2019 were averaged for each city.

We derived NDVI from Landsat 8 imagery at a 30-m spatial resolution. Cloud-masking procedures were applied to remove contaminated pixels, and annual NDVI values were calculated by averaging cloud-free observations from multiple images acquired throughout the year. The annual values for 2017–2019 were then averaged for each city to match the temporal coverage of the health and explanatory variables. NDVI represents vegetation greenness and photosynthetic activity, with higher values indicating greater vegetation density and overall vegetation health [41].

The TES is a composite index developed by American Forests that evaluates equity in access to urban tree canopy, with higher scores indicating a more equitable distribution of trees across neighborhoods [42]. It combines high-resolution land-cover data describing tree canopy distribution with demographic variables from the 2017–2021 American Community Survey, including the proportions of children, older adults, unemployed individuals, linguistically isolated residents, and dependent populations. The TES and its underlying methodology have recently been used as an equity-focused metric and framework in studies of housing and environmental justice, as well as urban violence prevention, particularly in the context of tree equity [[43], [44], [45]]. We averaged neighborhood-level TES values within each city's administrative boundary.

Mean annual temperature (MAT) and mean annual precipitation (MAP) were used as climate factors. We obtained city-level MAT and MAP for 2017–2019 from the Historical Monthly Weather Data of WorldClim Version 2.1 (2020) (www.worldclim.org), a widely used global climate database [46,47]. The climate data had a spatial resolution of 2.5 arcminutes (approximately 21 km^2^ per grid cell). Both variables were spatially averaged within each city's administrative boundary.

We included urban heat-island intensity and PM_2.5_ concentrations as urban environmental hazards representing urban heat exposure and air quality, respectively. The urban heat-island intensity data provided by American Forests [42] were generated by analyzing thermal extremes during the summer of 2022 using Landsat 8 Collection 2 Level-2 imagery obtained from the United States Geological Survey's EarthExplorer platform. This dataset quantitatively identifies areas within cities where land-surface temperature is relatively higher (heat-concentrated zones) or lower (relatively cool zones) than the citywide average [48]. We obtained annual PM_2.5_ concentrations from the global PM_2.5_ dataset developed by Wei et al. [48], which has a spatial resolution of 1 km. For each city, we averaged PM_2.5_ concentrations for 2017–2019 using the corresponding annual estimates from the complete 2017–2022 dataset. Urban heat-island intensity and PM_2.5_ values were spatially averaged within each city's geographic boundary.

To represent urbanization and socioeconomic conditions, we used four economic and social infrastructure variables: gross domestic product (GDP) per capita, poverty rate, access to healthcare, and population density. City-level GDP values were obtained from a gridded global dataset of total gross domestic product, modeled at five-year intervals from 1990 to 2020 by Kummu et al. [49], which provides spatially explicit GDP estimates at 1-km resolution. We calculated total city-level GDP by summing the values of all grid cells within each city boundary. GDP per capita was then calculated by dividing each city's total GDP in 2020 by the corresponding population estimate from the 2016–2020 American Community Survey (ACS) and was used as an indicator of city-level income. City-level poverty rates were also derived from the 2016–2020 ACS. We defined the poverty rate as the proportion of individuals aged 18–64 whose household incomes fall below the federal poverty threshold. Population density was calculated by dividing each city's total population by its land area in square kilometers.

We represented potential spatial access to healthcare using a 2019 global healthcare accessibility dataset, based in part on medical facility information from healthsites.io [50]. The dataset estimated travel time by motorized transport from each 1-km grid cell to the nearest medical facility, such as a hospital or clinic. We spatially averaged these travel-time estimates within each city boundary. Thus, higher values of the access-to-healthcare measure indicate longer travel times and lower accessibility. Although this measure does not directly capture healthcare utilization, quality of care, or affordability, travel-time-based accessibility metrics are widely used as proxies for potential spatial access in spatial epidemiology and health geography, particularly in large-scale ecological studies where standardized data on healthcare utilization are unavailable [[51], [52], [53]].

To capture structural social inequality, we used the proportion of people of color (POC) as an indicator of racial composition [27,29]. This variable was obtained from polygon data in the Tree Equity Score map developed by American Forests [42], which uses demographic information from the 2017–2021 ACS. The POC values were provided at the census block-group level and were spatially aggregated to the corresponding city boundaries. We performed all variable extraction and calculations using ArcGIS 10.5 (Esri, Redlands, California, United States).

Previous studies have associated these factors with various health indicators. However, most have examined individual factors or limited combinations of factors, and few have conducted comprehensive comparative analyses or examined their interrelationships [19,21]. The temporal coverage and exact years used for each variable are summarized in Supplementary Methods 1.1 and Table S2.

### 2.3 City stratification and grouping

**Fig. 1:**
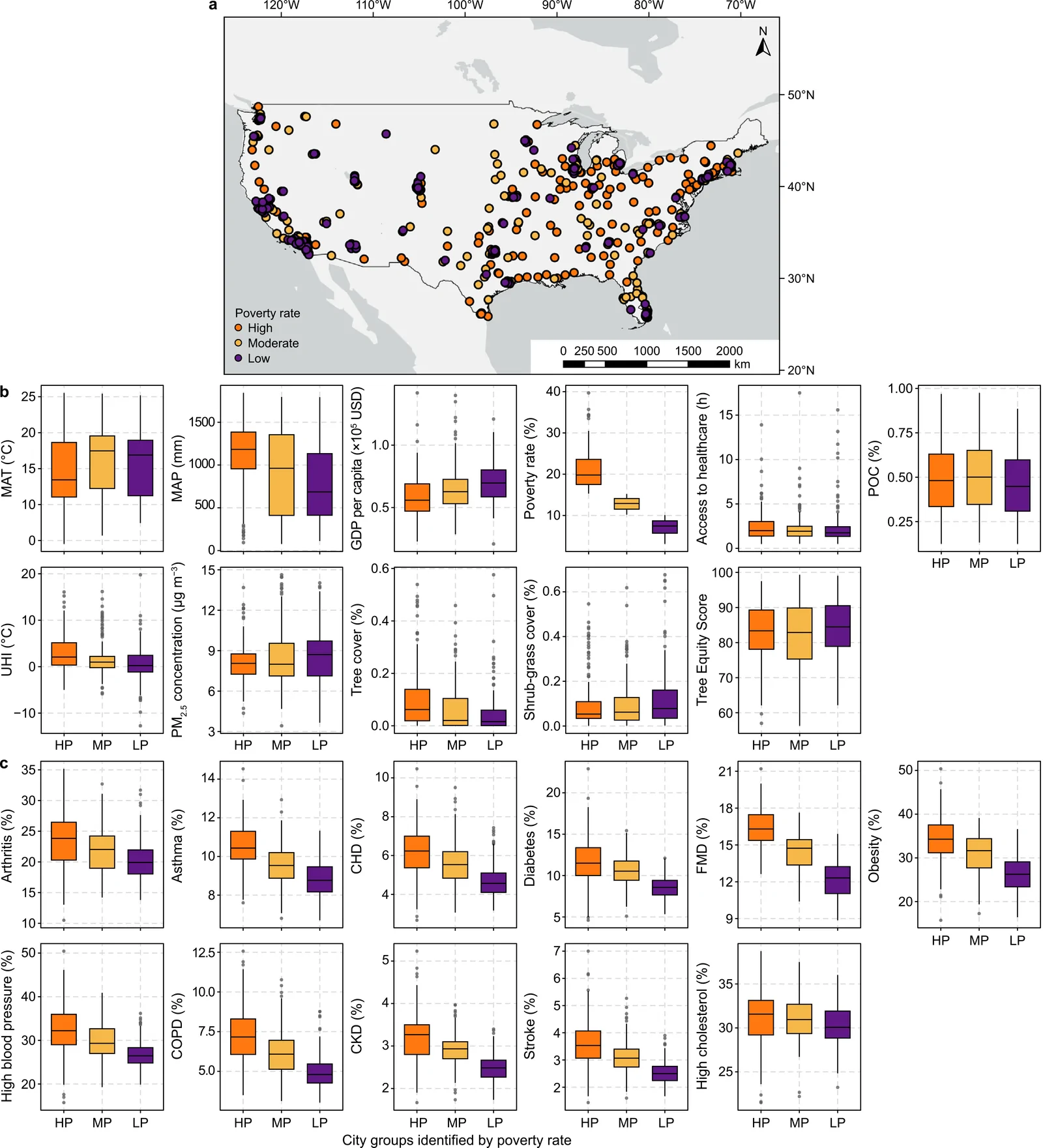
Geographic distribution and variation in explanatory variables and health outcomes across 497 cities in the United States grouped by poverty rate. a, Geographic distribution of cities classified into high-poverty (HP; 15.3–39.7%, *n* = 165), moderate-poverty (MP; 10.2–15.2%, *n* = 166), and low-poverty (LP; 3.1–10.1%, *n* = 166) groups. **b**–**c**, Distributions of explanatory variables (**b**) and health-outcome prevalence (**c**) across the three poverty groups. In the box plots, the center line indicates the median, boxes indicate the interquartile range, whiskers extend to 1.5 times the interquartile range, and points represent outliers. MAT, mean annual temperature; MAP, mean annual precipitation; GDP, gross domestic product; POC, proportion of people of color; UHI, urban heat-island intensity; PM_2.5_, fine particulate matter with an aerodynamic diameter of no more than 2.5 μm; CHD, coronary heart disease; FMD, frequent mental distress; COPD, chronic obstructive pulmonary disease; CKD, chronic kidney disease.

In addition to analyses using all cities combined, we conducted stratified analyses using two grouping schemes. First, cities were classified by poverty level as high poverty (15.3–39.7%), moderate poverty (10.2–15.2%), or low poverty (3.1–10.1%) based on poverty rate (Fig. 1; Supplementary Table S3). Second, city types were identified from multidimensional socioeconomic and environmental characteristics using Uniform Manifold Approximation and Projection (UMAP) followed by k-means clustering.

The poverty rate is a key indicator of socioeconomic inequality and is widely used to characterize structural differences in access to green space and public infrastructure, availability of healthcare services, and exposure to environmental hazards [54]. It is also commonly used in research on environmental justice and health disparities [54,55]. By stratifying cities by poverty level, we aimed to examine whether economic vulnerability modifies the associations between urban green space and health outcomes and to assess whether green space may help mitigate socioeconomic health disparities.

To capture multidimensional variation among cities beyond poverty alone, we applied a UMAP-based grouping approach. Rather than using geographic classifications, such as eastern versus western regions of the United States or state-level divisions, which may be subjective, this data-driven approach summarizes multidimensional climatic, socioeconomic, racial, and green-infrastructure characteristics. We generated the UMAP embedding using five nearest neighbors, a minimum distance of 0.1, and Euclidean distance, and then applied k-means clustering to the resulting coordinates. Based on the mean silhouette index, we initially identified six clusters and subsequently merged them into three city groups: Group A (101 cities), Group B (220 cities), and Group C (176 cities), to facilitate interpretation and ensure adequate sample sizes for subsequent analyses (Supplementary Table S3 and Fig. S2–S3). We implemented UMAP in R version 4.4.2 using the uwot package [56]. Additional details regarding the UMAP analysis, including clustering procedures and group classification, are provided in the Supplementary Methods.

We used permutational multivariate analysis of variance (PERMANOVA) with 9999 permutations to assess differences in predictor composition among the city groups. The analysis was based on a Euclidean distance matrix calculated from standardized explanatory variables and was implemented using the adonis2 function in the vegan package in R version 4.4.2. We assessed the homogeneity of multivariate dispersions using the betadisper function in the vegan package to assess whether differences in dispersion could contribute to the PERMANOVA results.

### 2.4 Statistical analysis

Before statistical analysis, variables were log- or square-root-transformed, as appropriate, to improve linearity and normality, and then standardized to reduce differences in measurement units and facilitate comparability. We first conducted correlation analyses to assess and minimize multicollinearity among variables for all cities and city groups (Supplementary Fig. S4). Population density was strongly correlated with access to healthcare and total green space proportion (|*r*| > 0.7), while total green space proportion was also strongly correlated with access to healthcare, shrub-grass cover, and population density (|*r*| > 0.7). NDVI also exhibited a strong positive correlation with MAP and tree cover (|*r*| > 0.7). We therefore excluded population density, total green-space proportion, and NDVI from the final analyses to reduce multicollinearity among the explanatory variables.

We fitted multivariable generalized least-squares (GLS) models with multimodel inference to quantify the relative importance of explanatory variables. We then applied piecewise structural equation modeling (pSEM) [57] to examine potential direct and indirect pathways linking green infrastructure, socioeconomic conditions, racial composition, environmental hazards, climate, and health-outcome prevalence (Supplementary Fig. S5). To account for spatial autocorrelation among cities, we incorporated an exponential spatial correlation structure into all GLS models used for both the multimodel inference and pSEM analyses. For pSEM, nonsignificant paths were removed based on d-separation tests, and models were compared using Fisher's C statistic, the associated *p-*value, and the Akaike information criterion. Detailed model specifications, selection procedures, and diagnostic results are provided in the Supplementary Methods and Supplementary Fig. S5.

We performed the relative-importance and pathway analyses for all cities combined and separately for the high-, moderate-, and low-poverty groups. Analyses based on the UMAP-based city typologies are presented as a supplementary analysis. We quantified the relative importance of each variable across the candidate model set by weighting its contribution in each model by that model's coefficient of determination (*R*^2^). Variable-level values were then aggregated into five explanatory-variable categories: climate, socioeconomic conditions, racial composition, environmental hazards, and green infrastructure.

We conducted the multimodel inference and pSEM analyses in R version 4.4.2 using the MuMIn [58] and piecewiseSEM [57] packages, respectively. Results for the remaining six outcomes, for which the models had lower explanatory power, are provided in Supplementary Information.

## 3 Results

### 3.1 Spatial distribution and baseline patterns of city groups

Across the 497 cities, high-poverty (HP), moderate-poverty (MP), and low-poverty (LP) cities were interspersed throughout the United States, with no clear regional separation (Fig. 1a). Compared with MP and LP cities, HP cities had lower GDP per capita, longer travel times to healthcare, and greater urban heat-island intensity. They also had higher precipitation and less shrub-grass cover (Fig. 1b). In contrast, LP cities had the highest GDP per capita, the shortest travel times to healthcare, and the greatest shrub-grass cover and TESs, but also the highest PM_2.5_ concentrations. MP cities generally showed intermediate socioeconomic conditions and green infrastructure characteristics. Median prevalence followed a consistent gradient across all 11 health outcomes, with the highest values in HP cities, the lowest values in LP cities, and intermediate values in MP cities (Fig. 1c).

The baseline characteristics of the UMAP-based city types are summarized in Supplementary Results 2.1.

### 3.2 Variance in health-outcome prevalence explained by the models

**Fig. 2:**
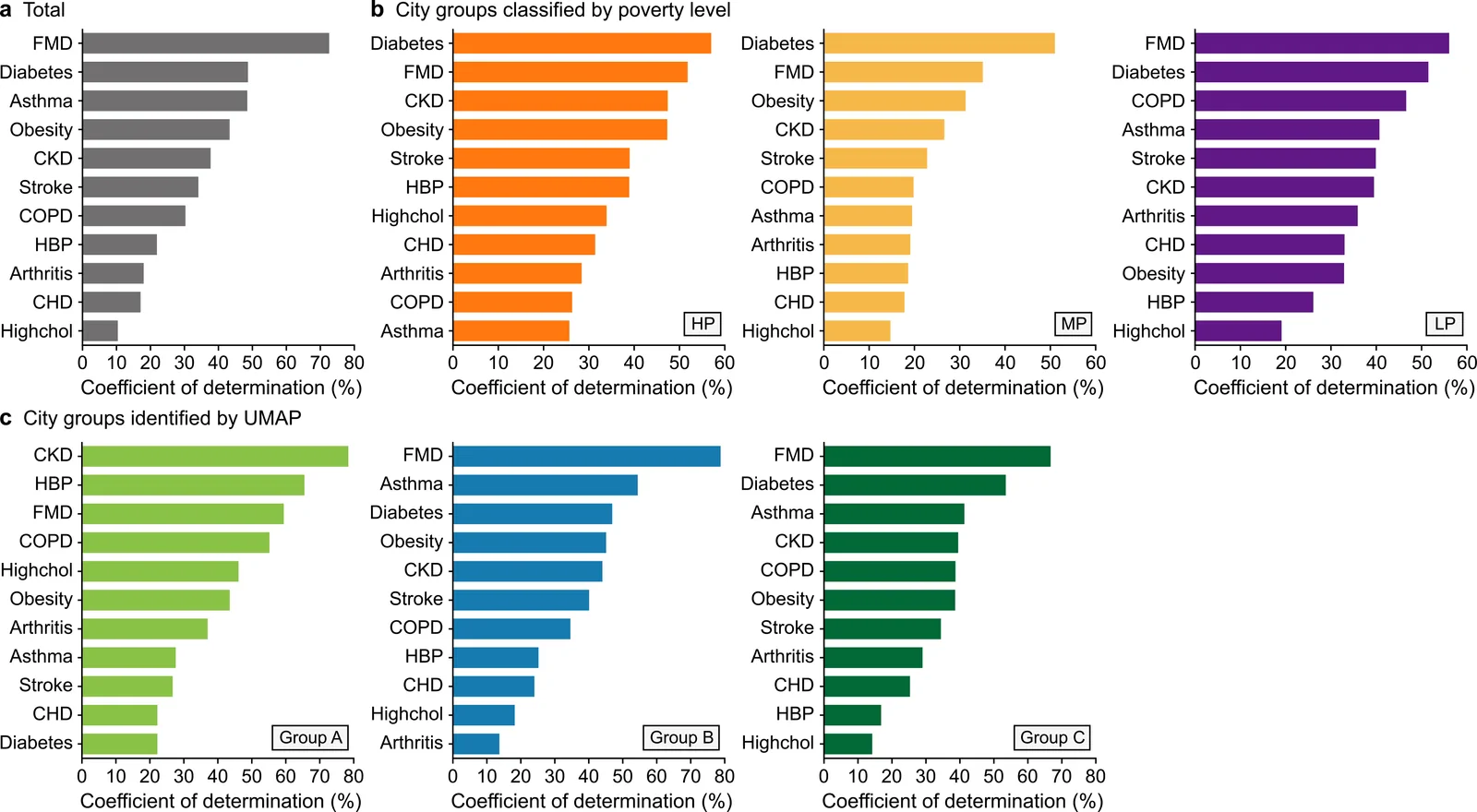
Explanatory power of multi-model inference analyses for 11 health outcomes across 497 cities in the United States and city groups. Ranked coefficients of determination for models fitted to all cities (**a**); cities classified as having high (HP), moderate (MP), or low (LP) poverty rates (**b**); and city groups A–C identified using Uniform Manifold Approximation and Projection (UMAP; **c**). UMAP-derived city groups were identified using UMAP, followed by k-means clustering based on multidimensional environmental, socioeconomic, racial, and green-infrastructure variables. Group A, B, and C included 101, 220, and 176 cities, respectively. Bar lengths indicate the percentage of variation explained by each model. HBP, high blood pressure; CHD, coronary heart disease; COPD, chronic obstructive pulmonary disease; CKD, chronic kidney disease; FMD, frequent mental distress; Highchol, high cholesterol.

Across all cities, the model for frequent mental distress had the greatest explanatory power (*R*^2^ = 0.726) and was the only model with an *R*^2^ exceeding 0.5 (Fig. 2a). For the remaining health outcomes, *R*^2^ ranged f from 0.104 for high cholesterol to 0.487 for diabetes.

In the poverty-stratified analyses, *R*^2^ exceeded 0.5 for diabetes in both HP and LP cities (HP, *R*^2^ = 0.570; LP, *R*^2^ = 0.515) and for frequent mental distress (HP, *R*^2^ = 0.518; LP, *R*^2^ = 0.561; Fig. 2b). In MP cities, only the model for diabetes exceeded this threshold (*R*^2^ = 0.510). Despite differences among city groups, diabetes, frequent mental distress, and stroke consistently ranked among the five health outcomes with the highest explained variance.

Among the UMAP-derived city groups, the model for frequent mental distress consistently showed explained variance exceeding 50% across all groups, while the models for CKD, high blood pressure, and COPD exceeded 50% in Group A, asthma in Group B, and diabetes in Group C (Fig. 2c; Supplementary Results 2.2).

### 3.3 Relative importance of factors associated with health-outcome prevalence

Across all cities, socioeconomic factors showed the highest relative importance for all health outcomes except arthritis, high blood pressure, diabetes, and high cholesterol (Table 1). At the individual-variable level, poverty rate had the greatest relative importance for the prevalence of frequent mental distress and asthma (Supplementary Fig. S6), whereas the proportion of POC had the greatest relative importance for diabetes. Although the relative importance of green infrastructure factors varied among health outcomes, they generally ranked as the second- or third-most important category of explanatory variables. Green infrastructure indicators, particularly TES and shrub-grass cover, were significantly negatively associated with the prevalence of several health outcomes.

**Table 1:** Relative importance and ranking of explanatory-variable categories across 11 health outcomes in 497 cities in the United States.

Explanatory-variable category	Arthritis	HBP	Asthma	CHD	COPD	Diabetes	High cholesterol	CKD	FMD	Obesity	Stroke	Mean rank
**All cities**												
Climate	7.39 (1)	9.25 (1)	0.00 (5)	4.82 (3)	8.97 (3)	9.25 (4)	5.00 (1)	3.50 (4)	3.33 (4)	0.70 (4)	8.20 (2)	2.91 ± 1.45
Socioeconomic conditions	3.18 (4)	3.46 (3)	30.47 (1)	6.32 (1)	10.90 (1)	9.30 (3)	2.20 (2)	14.95 (1)	38.64 (1)	15.36 (1)	12.05 (1)	1.73 ± 1.10
Racial composition	3.44 (3)	4.65 (2)	5.00 (3)	0.04 (5)	0.17 (5)	17.38 (1)	0.96 (4)	9.07 (2)	5.84 (3)	13.33 (3)	5.94 (4)	3.18 ± 0.25
Environmental hazards	0.13 (5)	1.37 (5)	1.59 (4)	0.21 (4)	1.18 (4)	3.02 (5)	0.12 (5)	1.10 (5)	1.36 (5)	0.16 (5)	1.39 (5)	4.73 ± 0.47
Green infrastructure	3.91 (2)	3.18 (4)	11.47 (2)	5.72 (2)	9.05 (2)	9.71 (2)	2.16 (3)	9.04 (3)	23.45 (2)	13.71 (2)	6.49 (3)	2.45 ± 0.69
Unexplained variance	81.95	78.09	51.47	82.89	69.73	51.34	89.56	62.34	27.38	56.74	65.93	-
**High-poverty cities**												
Climate	0.55 (3)	6.67 (4)	0.00 (5)	3.57 (3)	3.30 (3)	3.36 (4)	8.41 (3)	2.28 (4)	2.42 (4)	0.00 (5)	3.18 (5)	3.91 ± 0.83
Socioeconomic conditions	9.63 (2)	9.71 (2)	7.95 (2)	8.71 (2)	8.09 (2)	11.8 (3)	13.49 (1)	9.69 (3)	26.13 (1)	6.85 (3)	8.84 (2)	2.09 ± 0.70
Racial composition	0.00 (4)	9.09 (3)	5.18 (3)	2.89 (4)	0.03 (5)	19.98 (1)	0.85 (4)	15.18 (2)	0.00 (5)	18.59 (2)	8.19 (3)	3.27 ± 1.27
Environmental hazards	0.00 (4)	0.20 (5)	3.64 (4)	0.12 (5)	1.27 (4)	3.31 (5)	0.28 (5)	1.34 (5)	8.55 (3)	0.25 (4)	5.53 (4)	4.36 ± 0.67
Green infrastructure	18.24 (1)	13.25 (1)	8.90 (1)	16.15 (1)	13.65 (1)	18.57 (2)	10.91 (2)	18.88 (1)	14.69 (2)	21.58 (1)	13.28 (1)	1.27 ± 0.47
Unexplained variance	71.58	61.08	74.33	68.56	73.66	42.98	66.06	52.63	48.21	52.73	60.98	-
**Moderate-poverty cities**												
Climate	6.09 (1)	9.18 (1)	3.30 (3)	4.95 (2)	5.50 (2)	11.11 (3)	6.70 (1)	4.06 (4)	1.19 (4)	0.00 (5)	6.21 (2)	2.55 ± 1.37
Socioeconomic conditions	5.04 (3)	6.08 (2)	7.99 (1)	5.43 (1)	8.75 (1)	13.71 (2)	3.59 (2)	9.81 (1)	12.60 (1)	11.85 (1)	9.18 (1)	1.45 ± 0.69
Racial composition	5.92 (2)	1.18 (4)	3.05 (4)	3.06 (4)	0.25 (4)	15.89 (1)	3.36 (3)	6.05 (3)	8.39 (3)	9.68 (2)	2.24 (4)	3.09 ± 1.04
Environmental hazards	0.11 (5)	0.30 (5)	0.00 (5)	0.00 (5)	0.14 (5)	0.00 (5)	0.03 (5)	0.30 (5)	1.15 (5)	0.37 (4)	0.00 (5)	4.91 ± 0.30
Green infrastructure	1.99 (4)	1.83 (3)	5.11 (2)	4.32 (3)	5.18 (3)	10.29 (4)	1.02 (4)	6.36 (2)	11.73 (2)	9.38 (3)	5.22 (3)	3.00 ± 0.77
Unexplained variance	80.85	81.43	80.55	82.24	80.18	49.00	85.30	73.42	64.94	68.72	77.15	-
**Low-poverty cities**												
Climate	0.00 (5)	7.23 (2)	8.77 (3)	4.97 (4)	6.93 (4)	10.86 (2)	6.15 (2)	6.35 (2)	0.26 (5)	0.32 (4)	4.61 (4)	3.36 ± 1.21
Socioeconomic conditions	5.83 (3)	3.09 (4)	13.9 (1)	7.36 (3)	10.03 (3)	8.44 (3)	0.09 (4)	24.53 (1)	24.87 (2)	13.96 (2)	12.39 (2)	2.55 ± 1.04
Racial composition	16.95 (1)	4.25 (3)	7.52 (4)	9.85 (2)	11.97 (2)	8.23 (4)	6.13 (3)	3.45 (4)	1.22 (3)	0.00 (5)	6.45 (3)	3.09 ± 1.14
Environmental hazards	0.19 (4)	0.45 (5)	0.28 (5)	0.26 (5)	0.70 (5)	0.00 (5)	0.07 (5)	0.00 (5)	1.00 (4)	0.75 (3)	0.60 (5)	4.64 ± 0.67
Green infrastructure	12.96 (2)	11.11 (1)	10.23 (2)	10.61 (1)	17.02 (1)	23.97 (1)	6.63 (1)	5.17 (3)	28.79 (1)	17.9 (1)	15.89 (1)	1.36 ± 0.67
Unexplained variance	64.07	73.87	59.30	66.95	53.35	48.50	80.93	60.50	43.86	67.07	60.06	-

Cities were analyzed collectively and stratified into high-, moderate-, and low-poverty groups. Values indicate relative importance (%). Numbers in parentheses indicate rank within each health outcome, from 1 (highest) to 5 (lowest). Mean ranks are presented as mean ± standard deviation across the 11 outcomes. Unexplained variance was not ranked. HBP, high blood pressure; CHD, coronary heart disease; COPD, chronic obstructive pulmonary disease; CKD, chronic kidney disease; FMD, frequent mental distress.

When cities were grouped by poverty level, the results differed from those based on individual variables (Table 1 and Supplementary Fig. S7–S9). Nonetheless, the relative importance of green infrastructure factors was higher in the high- and low-poverty city groups than in the moderate-poverty group.

Analyses based on the UMAP-derived city groups showed broadly similar patterns of variable importance (Supplementary Results 2.3, Supplementary Table S4, and Supplementary Fig. S10–S12).

### 3.4 Structural associations between health-outcome prevalence and explanatory variables

**Fig. 3:**
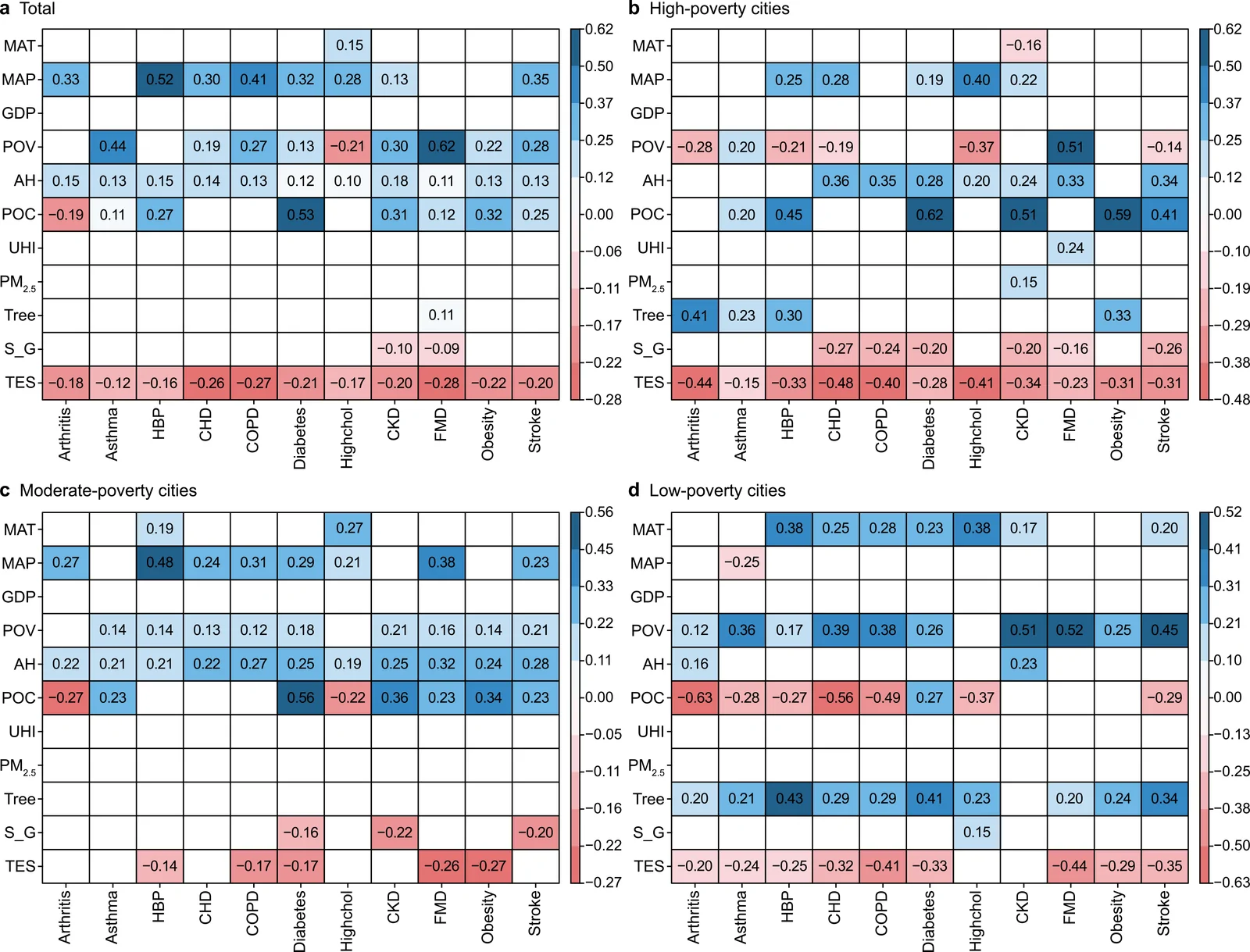
Structural associations between urban conditions and health-outcome prevalence across cities in the United States. Associations among climatic factors, socioeconomic conditions, racial composition, green infrastructure, environmental hazards, and health outcomes estimated using piecewise structural equation modeling (pSEM) for all cities (**a**), high-poverty cities (**b**), moderate-poverty cities (**c**), and low-poverty cities (**d**). Cell colors indicate the magnitude and direction of statistically significant direct associations according to the adjacent scales, and numerical values represent standardized direct path coefficients. White cells indicate no statistically significant direct associations. Detailed pSEM results, including indirect pathways, are provided in Supplementary Fig. S13–S26. MAT, mean annual temperature; MAP, mean annual precipitation; GDP, gross domestic product per capita; POV, poverty rate; AH, access to healthcare; POC, proportion of people of color; UHI, urban heat-island intensity; PM_2.5_, fine particulate matter with an aerodynamic diameter of no more than 2.5 μm; Tree, tree cover; S_G, shrub-grass cover; TES, Tree Equity Score. HBP, high blood pressure; CHD, coronary heart disease; COPD, chronic obstructive pulmonary disease; Highchol, high cholesterol; CKD, chronic kidney disease; FMD, frequent mental distress.

Across all cities, poverty rate and TES emerged as the variables most consistently associated with health-outcome prevalence (Fig. 3a; Supplementary Fig. S13–S14). Poverty rate showed positive direct associations with most health outcomes, with the largest standardized path coefficients (*β*) observed for frequent mental distress (0.62), asthma (0.44), CKD (0.30), stroke (0.28), COPD (0.27), and obesity (0.22). In contrast, TES exhibited significant negative associations across all health outcomes (*β* = −0.28 to −0.12), with the strongest associations observed for frequent mental distress (−0.28), COPD (−0.27), CHD (−0.26), obesity (−0.22), and diabetes (−0.21). Access to healthcare was positively associated with all health outcomes (*β* = 0.10–0.18). POC showed positive associations with several health outcomes, including diabetes (0.53), obesity (0.32), CKD (0.31), high blood pressure (0.27), and stroke (0.25), but was negatively associated with arthritis (−0.19). Among climatic factors, MAP was positively associated with arthritis, high blood pressure, CHD, COPD, diabetes, high cholesterol, CKD, and stroke, whereas MAT was positively associated with high cholesterol (0.15). Tree cover and shrub-grass cover exhibited limited disease-specific associations, and no significant direct associations were detected for urban heat-island intensity or PM_2.5_ concentrations.

In the high-poverty group, TES emerged as the most recurrent determinant of health outcome prevalence (Fig. 3b; Supplementary Fig. S15 and S16). TES exhibited significant negative associations with all health outcomes (*β* = −0.48 to −0.15), with the strongest associations observed for CHD (−0.48), arthritis (−0.44), high cholesterol (−0.41), COPD (−0.40), and CKD (−0.34). In contrast, POC showed positive associations with several health outcomes, particularly diabetes (0.62), obesity (0.59), CKD (0.51), high blood pressure (0.45), and stroke (0.41). Access to healthcare was positively associated with multiple health outcomes, including CHD, COPD, diabetes, high cholesterol, CKD, frequent mental distress, and stroke. Poverty rate exhibited disease-specific associations, showing positive relationships with asthma and frequent mental distress but negative associations with arthritis, high blood pressure, CHD, high cholesterol, and stroke. Among climatic factors, MAP showed positive associations with high blood pressure, CHD, diabetes, high cholesterol, and CKD, whereas MAT was negatively associated with CKD. Environmental hazard factors showed limited direct associations: Urban heat-island intensity was positively associated only with frequent mental distress (0.24), and PM_2.5_ concentration was positively associated only with CKD (0.15). Tree cover was positively associated with arthritis, high blood pressure, asthma, and obesity, whereas shrub-grass cover was negatively associated with CHD, COPD, diabetes, CKD, poor mental health, and stroke.

In the moderate-poverty group, access to healthcare and poverty rate showed positive associations with most health outcomes (Fig. 3c; Supplementary Fig. S17 and S18). TES exhibited negative associations with high blood pressure (*β* = −0.14), COPD (−0.17), diabetes (−0.17), frequent mental distress (−0.26), and obesity (−0.27). POC showed disease-specific positive associations, particularly with diabetes (0.56), CKD (0.36), and obesity (0.34). MAP was positively associated with multiple health outcomes, whereas shrub-grass cover was negatively associated with selected outcomes. No significant direct associations were detected for urban heat-island intensity, PM_2.5_ concentration, or tree cover.

In the low-poverty group, poverty rate showed positive associations with most health outcomes, whereas TES exhibited negative associations with multiple diseases, particularly frequent mental distress (*β* = −0.44), COPD (−0.41), stroke (−0.35), diabetes (−0.33), and CHD (−0.32) (Fig. 3d; Supplementary Fig. S19 and S20). POC was predominantly negatively associated with health outcomes, except for diabetes (0.27). MAT showed positive associations with several diseases, while MAP was negatively associated with asthma (−0.25). Tree cover was positively associated with multiple health outcomes, whereas shrub-grass cover showed a limited positive association with high cholesterol (0.15). No significant direct associations were detected for urban heat-island intensity or PM_2.5_ concentration.

Structural equation models stratified by UMAP-based city types showed broadly consistent patterns and are presented in Supplementary Results 2.4, Supplementary Table S4, and Supplementary Fig. S21–S26.

## 4 Discussion

Using harmonized data from 497 cities in the United States, we examined associations between green infrastructure, climate, environmental hazards, racial composition, and socioeconomic conditions and the prevalence of 11 chronic health outcomes. Three main findings emerged. First, the prevalence of most health outcomes increased with socioeconomic disadvantage, underscoring the important role of poverty, economic capacity, and healthcare access in urban health inequalities [59,60]. Second, higher TES and greater shrub-grass cover were consistently associated with lower disease prevalence, with particularly strong associations in high-poverty cities. Third, the relative importance and interaction pathways of these structural factors varied across city groups and poverty levels.

Across all cities, socioeconomic factors—poverty rate, GDP per capita, and access to healthcare—showed the strongest structural associations of health outcome prevalence. Higher poverty and lower levels of healthcare access were associated with higher prevalence of outcomes such as frequent mental distress, asthma, obesity, and CKD. Poverty and disease interact in a mutually reinforcing cycle: poverty functions both as a cause and a consequence of disease [61], and the World Health Organization has long referred to this phenomenon as “the diseases of poverty” [62]. In high-income countries such as the United States, poverty is a structural factor associated with socioeconomic stress and adverse household health and welfare outcomes [63,64]. Consistent with previous evidence, this study found that the poverty rate was the most influential predictor of mental health deterioration across all city groups. Although depression and anxiety are sometimes perceived as more frequent among economically privileged or socially advantaged individuals, these conditions account for approximately 8% of years lived with disability globally [65]. Within specific regions, individuals in the lowest income brackets are reported to be 1.5 to 3.0 times more likely to experience depression or anxiety than those in high-income groups [65]. These disparities are shaped by multiple social stressors, including poor physical health, trauma, exposure to violence and crime, and diminished social status [65,66]. In addition, low-income households living in substandard housing may be disproportionately exposed to environmental stressors such as pollution, extreme temperatures, and poor sleep environments, which may exacerbate mental health conditions and lifestyle-related conditions such as asthma and CKD. These findings indicate that poverty is not merely an economic deficit but a multidimensional factor associated with both mental and physical health. Reducing urban health inequalities may therefore require improvements in medical infrastructure alongside interventions, such as enhancing residential conditions, within a broader ecological and social framework.

Our findings identify green infrastructure as a key factor in reducing health-outcome prevalence across cities and city groups. Research examining the relationships between urban green space, human health, and disease has steadily expanded in recent years [[67], [68], [69]]. Although greater access to green spaces positively influences the health of urban residents, this relationship is not universal, and negative outcomes have also been reported in specific contexts [70]. Given the diverse socioeconomic and environmental conditions across cities, identifying whether and how green infrastructure and accessibility influence health, both positively and negatively, is therefore essential for planning healthy and sustainable urban environments. In this context, our study showed that health-outcome prevalence tends to decrease as equity in access to urban green space improves, as reflected by higher TES, and for several outcomes, as the proportion of shrub-grass cover increases. Across all cities, for health outcomes in which TES was significantly associated with prevalence, a 5-point increase in TES was associated with a 2.72–35.34% lower prevalence, corresponding to 624–1405 fewer cases per 100,000 population. This inverse association between TES and health-outcome prevalence was more pronounced in high-poverty cities, although its magnitude varied by health outcome and city group (Supplementary Table S5).

Urban green spaces can support mental and physical health through several pathways [18]. Greater green-space density may reduce exposure to noise, heat, and air pollution [71], while creating more comfortable environments for outdoor recreation and physical activity [72]. Contact with nature may support psychological and physiological restoration, thereby reducing the risk of frequent mental distress [73].

Although the United States is among the world's wealthiest countries, its poverty rate remains relatively high compared with those of other high-income democratic countries [74,75]. These structural vulnerabilities contribute to persistent differences in disease burden [76,77]. Among low-income groups, infectious diseases, nutritional deficiencies, and a wide range of chronic health conditions often impose a disproportionate burden. Poverty is also associated with multiple adverse health determinants, including limited access to healthcare, unhealthy environmental conditions, psychosocial stress, and a higher prevalence of some health-risk behaviors. Therefore, poverty functions not merely as an indicator of economic deprivation but as a broader socioeconomic metric that reflects multiple pathways through which health inequalities may arise and persist [62].

Green infrastructure had greater relative importance in explaining health-outcome prevalence in both high- and low-poverty city groups than in moderate-poverty cities. This finding is potentially relevant for policy because green infrastructure may be more amenable to intervention than structural socioeconomic conditions and may help reduce disease burden across the socioeconomic spectrum. From an environmental-justice perspective, socioeconomically disadvantaged communities are often disproportionately exposed to environmental hazards, including heat stress, air pollution, and limited access to health-promoting green infrastructure. Therefore, improving equitable access to urban green spaces may provide greater health benefits in vulnerable communities, highlighting the importance of equity-focused urban greening strategies [55].

However, the pathways underlying the observed associations between green space and health outcomes appear to differ between these high- and low-poverty cities. In high-poverty cities, green space showed both direct and indirect associations with health outcomes by mitigating urban environmental hazards, including PM_2.5_ concentration and urban heat-island intensity, which have been associated with conditions such as COPD and CKD. By contrast, in low-poverty cities, green infrastructure showed stronger direct associations with health-outcome prevalence. This may be because low-poverty cities have greater healthcare accessibility, better living conditions, and more social resources, which may enable green spaces to support health more directly through pathways related to physical activity, psychological restoration, and social interaction [72,73].

In cities with moderate poverty levels, economic and racial factors showed stronger associations with health-outcome prevalence than green infrastructure. One possible explanation is that moderate-poverty cities often exhibit substantial demographic heterogeneity and relatively high proportions of POC. Previous studies have suggested that racial composition may reflect broader structural and contextual conditions, including residential segregation, differential environmental exposures, healthcare accessibility, and neighborhood inequalities [78,79]. Therefore, the stronger associations observed for POC in these cities should be interpreted as reflecting structural social conditions rather than direct biological effects. In addition, the relatively lower importance of green infrastructure may indicate that health outcomes in moderate-poverty cities are shaped by a broader set of interacting social, economic, and environmental factors. However, our analysis did not include individual-level variables such as diet, physical activity, healthcare utilization, or chronic disease management; the mechanisms underlying these patterns remain uncertain and warrant further investigation [62]. As a result, the associations between green infrastructure and health outcomes may be diminished or overshadowed. Moreover, the city-level approach used in this study may not fully capture the complex relationships between green infrastructure and health outcomes at finer spatial scales. Future studies incorporating individual-level health data and fine-scale environmental information are therefore needed to better understand the mechanisms underlying these associations.

The observed associations may arise through multiple pathways, including heat mitigation, reduced exposure to air pollution, promotion of physical activity, enhanced social interaction, and psychosocial restoration. The relative importance of these pathways is likely to vary across cities according to their socioeconomic conditions, environmental stressors, and access to health-supporting resources. Our findings therefore suggest that the associations between green infrastructure and health are context-dependent rather than uniform across cities.

Although the models explained substantial variation in several health outcomes, relatively low explanatory power was observed for outcomes such as stroke, COPD, high blood pressure, and high cholesterol. These conditions are influenced by factors such as smoking, alcohol consumption, diet, medication adherence, healthcare utilization, and genetic predisposition, many of which were not included in the present city-level analysis. Measurement uncertainty and the omission of potentially important individual-level determinants may have contributed to the lower explanatory power observed for these outcomes. Environmental and socioeconomic variables alone may be insufficient to explain variation in these health outcomes across cities. Nevertheless, the overall patterns observed across multiple health outcomes indicate that green infrastructure remains an important component of urban health promotion, particularly when implemented in ways that account for local socioeconomic and environmental conditions.

Equity-focused urban greening should be considered as one component of a broader strategy to address structural determinants of health. Policy implications may differ across socioeconomic contexts. In high-poverty cities, interventions could prioritize tree-canopy expansion and equitable access to neighborhood green spaces in heat-vulnerable and environmentally underserved areas. In moderate-poverty cities, green initiatives could be integrated with efforts to improve healthcare accessibility and reduce neighborhood-level social inequalities. In low-poverty cities, further benefits might be achieved by improving connections among existing green spaces. These findings suggest that urban greening policies should move beyond a one-size-fits-all approach and instead adopt strategies tailored to local socioeconomic conditions and environmental needs.

Group C provided further evidence that racial composition should be interpreted within its broader structural context. The UMAP-derived groups showed distinct geographical concentrations: Group A primarily represented cities in the southeastern United States, Group B was dominated by cities in the northern United States, and Group C mainly consisted of cities in the southwestern United States. This pattern suggests that the association between racial composition and health outcomes may vary across different socioeconomic and environmental contexts. Despite having a relatively high proportion of people of color, Group C exhibited relatively low poverty rates. This pattern suggests that racial composition alone may not adequately explain patterns of health-outcome prevalence. Previous studies have shown that health disparities associated with racial and ethnic composition are often shaped by broader structural and contextual factors, including socioeconomic conditions, healthcare accessibility, environmental exposures, and experiences of discrimination [80,81]. The pattern observed in Group C may therefore reflect differences in these contextual conditions rather than racial composition itself.

Social cohesion, community resources, and neighborhood characteristics may influence population health, but these variables were not directly measured in this study. Consequently, the mechanisms underlying the observed pattern cannot be determined and should be interpreted with caution. These findings nevertheless highlight the importance of considering racial composition within its broader social, economic, and environmental context when examining urban health inequalities. Future urban health and environmental policies should therefore move beyond treating racial and socioeconomic characteristics as isolated factors and instead address the broader structural determinants of health that shape outcomes across communities.

Several limitations should be acknowledged. First, the analysis relies on cross-sectional ecological data with imperfect temporal alignment among variables, and the use of remotely sensed and publicly available datasets introduces measurement and spatial-scale uncertainties. In particular, urban heat-island intensity was measured in 2022, whereas the health outcomes covered 2017–2019. Although urban heat-island intensity is largely influenced by relatively stable urban structural characteristics, such as impervious surfaces, building density, urban form, and green distribution, and may therefore vary less over short time periods [82,83], this temporal mismatch remains a source of uncertainty. Second, aggregating all variables to the city level may obscure substantial within-city spatial heterogeneity in green space distribution, heat exposure, air pollution, and socioeconomic conditions. As a result, important within-city disparities may have been averaged out, limiting the ability to capture fine-scale variation in environmental exposures and health outcomes. Third, the green infrastructure indicators used in this study primarily captured the quantity and equity of urban green spaces and did not explicitly account for ecological characteristics such as biodiversity, vegetation structure, habitat connectivity, ecological quality, or management quality. Ecological and experiential pathways dimensions of green infrastructure that may be relevant to health were therefore incompletely represented. Furthermore, the analysis did not include individual-level behaviors such as smoking, alcohol consumption, diet, and physical activity, and potentially important contextual factors, including municipal health policies and urban governance characteristics,. The absence of these variables may have resulted in residual confounding and could have influenced both the direction and magnitude of the observed associations. Genetic susceptibility and other individual-level factors may also account for some of the unexplained variation in health outcomes.

Future research should address these limitations by incorporating genetic susceptibility and potential gene–environment interactions, linking small-area or individual-level health data with fine-scale environmental exposure data, and applying longitudinal or multilevel approaches. Spatially explicit datasets describing biodiversity, vegetation structure, habitat connectivity, ecological quality, management characteristics, and perceived safety across multiple spatial scales, from national to global levels, would enable evaluation of green infrastructure beyond simple measures of quantity and equity. Expanding analyses to include these qualitative dimensions, together with indicators of social and institutional environments, health-risk behaviors, and other natural environments such as blue spaces, would provide a more comprehensive understanding of how environmental and socioeconomic conditions interact to shape urban health inequalities and influence health outcomes.

## 5 Conclusion

This study examined the prevalence of 11 major health outcomes across 497 cities in the United States while simultaneously accounting for urban green infrastructure, climate conditions, socioeconomic factors, racial composition, and environmental hazards. By integrating multidimensional indicators, including green-space quantity and equity, climate variables, urban heat-island intensity, air pollution, and socioeconomic conditions, within a single analytical framework, and by stratifying cities according to their characteristics and poverty levels, we found that associations among green space, socioeconomic indicators, and health-outcome prevalence vary substantially depending on urban context. These findings may have implications for environmental justice and urban public health planning. In particular, the health relevance of green infrastructure was strongly influenced by socioeconomic context, suggesting that the potential health benefits of urban greening are not distributed uniformly across cities. Urban greening policies should therefore move beyond a one-size-fits-all approach and adopt context-specific strategies that address local environmental conditions, socioeconomic vulnerability, and inequities in access to green infrastructure.

## CRediT authorship contribution statement

**Min-Ki Lee:** Writing – original draft, Methodology, Formal analysis, Data curation, Visualization. **Chang-Bae Lee:** Conceptualization, Funding acquisition, Methodology, Project administration, Supervision, Writing – review & editing. **Sungyong You:** Conceptualization, Funding acquisition, Methodology, Project administration, Supervision, Writing – review & editing. **Stephen J. Freedland:** Writing – review & editing, Supervision. **Michael R. Freeman:** Writing – review & editing, Supervision.

## Data availability

The original data used in this study are provided in Supplementary Table S1. Additional data are available from the corresponding author, Prof. Chang-Bae Lee, PhD, on reasonable request.

## Declaration of competing interest

The authors declare that they have no known competing financial interests or personal relationships that could have appeared to influence the work reported in this paper.
