# Supplementary material for: Poverty-modulated tree equity shapes health burdens across U.S. cities
Source: Environ Sci Ecotechnol. 2026 Sep 3;33:100758. doi: 10.1016/j.ese.2026.100758 (PMC13578987; doi:10.1016/j.ese.2026.100758)
Supplement: Supplementary file 1 [file mmc1.docx]

**Supplementary Information**

**Poverty-modulated tree equity shapes health burdens across U.S. cities**

Min-Ki Lee^a^, Chang-Bae Lee^a,b,*^, Sungyong You^c,d,e,*^, Stephen Freedland^c^, Michael Freeman^c,f^

^a^Department of Forest Resources, Kookmin University, Seoul 02707, Republic of Korea

^b^Department of Climate Convergence Technology, Kookmin University, Seoul 02707, Republic of Korea

^c^Department of Urology, Cedars-Sinai Medical Center, Los Angeles, CA 90048, USA

^d^Department of Computational Biomedicine, Cedars-Sinai Medical Center, Los Angeles, CA 90048, USA

^e^Department of Biopharmaceutical and Regulatory Science, School of Pharmacy, Sungkyunkwan University, Suwon 16419, Republic of Korea

^f^Department of Biomedical Sciences, Cedars-Sinai Medical Center, Los Angeles, CA 90048, USA

Correspondence: Chang-Bae Lee (kecolee@kookmin.ac.kr), Sungyong You (Sungyong.You@cshs.org)

**1. Supplementary Methods**

**1.1.** Spatiotemporal resolution of the data used in the analysis

The explanatory variables used in this study—green infrastructure factors, climate factors, socioeconomic factors, racial composition and urban environmental hazard factors—as well as the dependent variable, health outcome prevalence, differ in their temporal resolutions (Table S2), which required careful consideration in the analysis. Among these variables, health outcome prevalence, tree and shrub-grassland cover, climate factors (i.e., mean annual temperature and mean annual precipitation), PM_2.5_, and the poverty rate were consistently available for the period 2017–2022. By contrast, tree equity score and the proportion of people of color were available only for 2017–2021, GDP for 2020, access to healthcare for 2019, and urban heat island intensity data for 2022. To minimize loss of temporal information while maximizing overlap among variables, we used data from the period with the greatest common coverage, 2017–2019, for the primary analysis. An exception was made for urban heat island intensity, for which only 2022 data were available; therefore, the 2022 values were included in the analysis.

**1.2.** City stratification and grouping

Cities differ in their configurations of green infrastructure, climatic conditions, socioeconomic status, and environmental hazards, making it difficult to fully explain variations in health outcome prevalence using city-level analyses alone (Kabisch et al., 2015; Cox et al., 2018). Therefore, to capture broader intrinsic characteristics and levels of socioeconomic vulnerability, we conducted an additional grouping analysis using UMAP (Uniform Manifold Approximation and Projection; McInnes et al., 2018), independent of the poverty-level classification described in the main text.

We applied UMAP using 11 explanatory variables, including green infrastructure factors, to identify multidimensional environmental patterns across cities. Through this clustering process, cities were grouped into three distinct clusters (Figs S2 and S3). This approach allowed us to classify cities not by single indicators but by combined profiles of urban characteristics (Andersson et al., 2021; Rodrigues et al., 2025), and to examine how health outcome prevalence varies across contrasting urban contexts.

**1.3.** Statistical analysis

To classify and group cities based on their multidimensional characteristics, principal component analysis (PCA) was performed using the standardized set of 11 explanatory variables. The principal component scores for each city were then used as input variables to UMAP to embed cities into a two-dimensional latent space. UMAP is a manifold-learning technique for dimensionality reduction grounded in Riemannian geometry and algebraic topology. It is widely used in machine learning because of its computational efficiency for high-dimensional embedding (McInnes et al., 2018). UMAP hyperparameters were set to n_neighbors = 5, min_dist = 0.1, and Euclidean distance as the metric.

K-means clustering was subsequently applied to the resulting two-dimensional UMAP coordinates (umap_2d). The optimal number of clusters was determined by varying K from 2 to 10 and selecting the value that yielded the highest mean silhouette index, which resulted in six initial clusters. For interpretability and geographic coherence, as well as to ensure adequate sample sizes, Clusters 1 and 4 were combined into Group A (101 cities), Clusters 2 and 5 into Group B (220 cities), and Clusters 3 and 6 into Group C (176 cities) (Figures S2 and S3). These grouped city types were then used in the subsequent analyses of health outcome prevalence.

To assess the relative importance of explanatory variables for the prevalence of each health outcome, we used multi-model inference tests based on model-averaging (Legendre and Legendre, 2012). Before model evaluation, variance inflation factors (VIFs) were calculated to assess multicollinearity in the multiple regression models. All VIF values were below 3, indicating that multicollinearity among explanatory variables was unlikely to bias model results. To account for spatial autocorrelation, we fitted generalized least squares models that incorporated each city’s geographic coordinates. To prevent model overfitting and ensure robust averaged estimates, only models with ΔAIC values ≤ 2 were included in the averaging procedure (Legendre and Legendre, 2012; García-Palacios et al., 2018). Relative importance values were then calculated by summing the absolute standardized coefficients (β) of each variable across the selected models and dividing each variable’s summed coefficient by the total, thereby deriving relative importance at both variable and factor level.

In addition, to examine causal linkages among explanatory variables, we constructed a piecewise structural equation model (pSEM; Lefcheck, 2016) based on a conceptual model (Figure S5). Using pSEM, we evaluated both the direct and indirect effects of green space, climate, environmental hazards, racial composition, and socioeconomic factors on health outcome prevalence. For optimal model selection, we first built a full model containing all plausible paths between the prevalence of health outcomes and the explanatory variables and then iteratively removed nonsignificant paths using d-separation tests to identify the best-fitting model (Lefcheck, 2016; Lee et al., 2023). Model fit for the final pSEM was evaluated using Fisher’s C statistic, p-value, and AIC.

Because the primary objective of this study was to evaluate the relationships between environmental, socioeconomic, and health variables at the national scale and across poverty contexts, the relative importance of explanatory variables and their causal structures were analyzed for the entire dataset and for the poverty-based city groups (HP, MP, and LP). UMAP-based city typologies were included as a supplementary analysis to assess the robustness of the findings under an alternative city classification scheme. Therefore, only variable importance results for the UMAP-based groups are presented in Table S3. To facilitate comparisons among city groups, relative importance values obtained from MuMIn-based multi-model inference were weighted by the coefficient of determination (R²) of the corresponding health outcome model. The weighted values were then aggregated into five explanatory-variable categories (climate, socioeconomic conditions, racial composition, environmental hazards, and green infrastructure) to obtain category-level relative importance.

All UMAP, multi-model inference, and pSEM analyses were conducted in R version 4.4.2 (R Core Team, 2024) using the packages uwot (Melville, 2025), MuMIn (Bartoń, 2025), and piecewiseSEM (Lefcheck, 2016), respectively.

**2. Supplementary Results**

**2.1.** Results of spatial distribution of cities and baseline patterns by poverty level and UMAP-based city types

Uniform Manifold Approximation and Projection (UMAP) applied to the environmental and socioeconomic variables identified six clusters of cities, which we aggregated into three broader city types (Figs S2 and S3). Group A cities, located mainly in the south-eastern U.S., were the warmest and wettest, with the highest mean annual temperature and precipitation. They had the lowest GDP per capita among the three types, relatively highest poverty, the highest access time to healthcare, an intermediate proportion of people of color, the highest tree cover, intermediate shrub–grass cover, intermediate PM_2.5_ concentrations, intermediate urban heat-island intensity, and the highest Tree Equity Scores (TES). Group B cities formed a band across the northern U.S. They had the coolest climates, high (but not maximal) precipitation, intermediate GDP per capita and poverty rates, the lowest proportion of people of color, the strongest urban heat-island intensity, the lowest PM_2.5_ concentrations, moderate tree cover, the lowest shrub–grass cover, and intermediate TES. Group C cities were concentrated in the south-western U.S. They were warm and dry, with the lowest precipitation, GDP per capita similar to Group B, the lowest poverty levels, the highest proportion of people of color, moderate travel times to healthcare, the highest PM_2.5_ concentrations, very low tree cover, the highest shrub–grass cover, and the lowest TES.

For most health outcomes, prevalence decreased monotonically from Group A to Group C, with Group B showing intermediate values. In contrast, diabetes and CKE exhibited distinct patterns, with the lowest prevalence observed in Group B.

**2.2.** Results of explanatory power of factors for health outcome prevalence

Among the city groups identified through UMAP-based clustering, frequent mental distress consistently exhibited explained variances exceeding 50% across all groups (Group A: 59.4%, Group B: 78.8%, and Group C: 66.7%). In Group A, chronic kidney disease (78.4%), high blood pressure (65.5%), and chronic obstructive pulmonary disease (56.2%) also exhibited explained variances exceeding 50%. In Group B, asthma exhibited an explained variance of 54.4%, whereas in Group C, diabetes exhibited an explained variance of 53.5% (Fig 2c).

**2.3.** Results of relative importance of factors for health outcome prevalence

The UMAP-based clustering analysis revealed that green infrastructure was the most important factor influencing disease prevalence in Groups A and B, whereas socioeconomic factors were the dominant determinants of health outcomes in Group C (Table S4 and Figs S10–S12). Although some group- and outcome-specific differences were observed, socioeconomic factors, racial composition, and green-infrastructure variables consistently emerged as key determinants of health outcome prevalence.

**2.4.** Results of direct and indirect associations between health outcome prevalence and explanatory variables

The pSEM results for Group A, TES, and poverty rate emerged as the variables most consistently associated with health outcome prevalence (Figs. S21−S22). TES exhibited significant negative direct associations with all health outcomes except asthma, with particularly strong associations with coronary heart disease (CHD; β = −0.56), chronic obstructive pulmonary disease (COPD; β = −0.52), chronic kidney disease (CKD; β = −0.49), high blood pressure (β = −0.48), diabetes (β = −0.45), arthritis (β = −0.44), and stroke (β = −0.41). In contrast, poverty rate was positively associated with frequent mental distress (FMD; β = 0.81), asthma (β = 0.38), COPD (β = 0.29), stroke (β = 0.27), CKD (β = 0.25), CHD (β = 0.22), and obesity (β = 0.17). POC was positively associated with diabetes (β = 0.50), obesity (β = 0.48), asthma (β = 0.37), high blood pressure (β = 0.34), stroke (β = 0.31), and CKD (β = 0.24). On the other hand, POC showed a negative relationship with high cholesterol (β = −0.37) and CHD (β = −0.22). Additionally, access to healthcare does not affect any health outcome. Climate and environmental hazard factors generally exhibited weaker and more disease-specific effects. MAT was negatively associated with asthma and arthritis, whereas MAP was positively associated with both. PM_2.5_ was positively associated with kidney disease, high cholesterol, stroke, CHD, and diabetes. Tree cover was positively associated with FMD. Furthermore, TES was negatively associated with environmental hazard factors, particularly PM_2.5_, thereby exhibiting both direct associations and indirect pathway-based associations through environmental hazards.

In Group B, TES emerged as the most consistent determinant of the prevalence of health outcomes (Figs. S23−S24). TES exhibited significant negative associations with all health outcomes, for COPD (β = −0.34), CHD (β = −0.32), diabetes (β = −0.29), stroke (β = −0.28), high cholesterol (β = −0.28), obesity (β = −0.27), arthritis (β = −0.24), CKD (β = −0.24), FMD (β = −0.24), high blood pressure (β = −0.23), and asthma (β = −0.12). In contrast, poverty rate showed positive associations with most health outcomes, with effects on FMD (β = 0.66), asthma (β = 0.51), CKD (β = 0.29), COPD (β = 0.25), stroke (β = 0.25), obesity (β = 0.23), and CHD (β = 0.14). However, poverty rate had a negative effect on high cholesterol (β = −0.30). POC was positively associated with diabetes (β = 0.46), obesity (β = 0.32), CKD (β = 0.28), high blood pressure (β = 0.28), stroke (β = 0.24), asthma (β = 0.14), and FMD (β = 0.07). Access to healthcare showed a positive relationship with FMD, asthma, obesity, CKD, COPD, high blood pressure, CHD, high cholesterol, and arthritis. MAT was positively associated with FMD, COPD, and high cholesterol, whereas PM_2.5_ was positively associated with diabetes, CKD, stroke, and CHD. Tree cover showed positive associations with diabetes, CKD, stroke, COPD, CHD, high cholesterol, and arthritis, whereas shrub-grass cover was negatively associated with FMD, diabetes, CKD, stroke, COPD, CHD, and high cholesterol. Furthermore, TES was negatively associated with environmental hazard factors, particularly PM_2.5_ and urban heat-island intensity, thereby influencing health outcomes both directly and indirectly through reductions in environmental hazards.

In Group C, TES were the most consistent determinants of the prevalence of health outcomes (Figs. S25−S26). TES showed a significant negative correlation with almost all health outcomes, excluding CKD. Poverty rate showed positive associations with most health outcomes, with particularly strong effects on FMD (β = 0.63), asthma (β = 0.48), CKD (β = 0.47), stroke (β = 0.44), COPD (β = 0.44), CHD (β = 0.34), diabetes (β = 0.27), obesity (β = 0.20), and high blood pressure (β = 0.18). POC showed disease-specific associations, being positively associated with diabetes (β = 0.47), CKD (β = 0.20), and obesity (β = 0.11), while showing negative associations with arthritis (β = −0.54), COPD (β = −0.29), high cholesterol (β = −0.25), CHD (β = −0.19), and asthma (β = −0.18). In contrast, Climate and environmental hazard factors generally exhibited weaker and disease-specific effects. MAT was positively associated with high cholesterol (β = 0.25), obesity (β = 0.24), FMD (β = 0.17), and diabetes (β = 0.12), whereas PM_2.5_ showed negative associations with FMD and high cholesterol. Tree cover was negatively associated with CKD (β = −0.27), while shrub-grass cover was negatively associated with FMD (β = −0.16). In addition, access to healthcare showed a positive relationship with all health outcomes except high cholesterol.

**References**

Andersson, E., Haase, D., Anderson, P., Cortinovis, C., Goodness, J., Kendal, D., ... & Wellmann, T. (2021). What are the traits of a social-ecological system: Towards a framework in support of urban sustainability. *NPJ Urban Sustainability*, *1*(1), 14.

Bartoń, K. (2022). MuMIn: multi-model inference. *R package version 1.46.0.*

Cox, D. T., Shanahan, D. F., Hudson, H. L., Fuller, R. A., & Gaston, K. J. (2018). The impact of urbanisation on nature dose and the implications for human health. *Landscape and Urban Planning*, *179*, 72-80.

García-Palacios, P., Gross, N., Gaitán, J., & Maestre, F. T. (2018). Climate mediates the biodiversity–ecosystem stability relationship globally. *Proceedings of the National Academy of Sciences USA*, *115*(33), 8400-8405.

Kabisch, N., Qureshi, S., & Haase, D. (2015). Human–environment interactions in urban green spaces—A systematic review of contemporary issues and prospects for future research. *Environmental Impact Assessment Review*, *50*, 25-34.

Lee, M. K., Lee, Y. J., & Lee, C. B. (2023). Landscape and marine environmental factors jointly regulate the intertidal species richness and community structure in the islands of South Korea. *Diversity*, *15*(7), 826.

Lefcheck, J. S. (2016). piecewiseSEM: Piecewise structural equation modelling in r for ecology, evolution, and systematics. *Methods in Ecology and Evolution*, *7*(5), 573-579.

Legendre, P., & Legendre, L. (2012). Numerical Ecology, third English ed. *Developments in Environmental Modelling. Elsevier, Amsterdam*, *1006*.

McInnes, L., Healy, J., & Melville, J. (2018). Umap: Uniform manifold approximation and projection for dimension reduction. *arXiv preprint arXiv:1802.03426*.

Melville, J. (2025). Package uwot: the Uniform Manifold Approximation and Projection method for dimensionality reduction. R package version 0.2.4.

Rigolon, A., Browning, M. H., McAnirlin, O., & Yoon, H. (2021). Green space and health equity: a systematic review on the potential of green space to reduce health disparities. *International Journal of Environmental Research and Public Health*, *18*(5), 2563.

Rodrigues, C., Veloso, M., Alves, A., & Bento, C. L. (2025). Socioeconomic and functional zoning characterization in a city: A clustering approach. *Cities*, *163*, 106023.

Wolch, J. R., Byrne, J., & Newell, J. P. (2014). Urban green space, public health, and environmental justice: The challenge of making cities ‘just green enough’. *Landscape and Urban Planning*, *125*, 234-244.

Table S2. Temporal coverage of explanatory and response variables used in this study and the number of available datasets by year. Gray-filled cells indicate years for which data were available.

| Indices Years | 2017 | 2018 | 2019 | 2020 | 2021 | 2022 |
| --- | --- | --- | --- | --- | --- | --- |
| Mean annual temperature |  |  |  |  |  |  |
| Mean annual precipitation |  |  |  |  |  |  |
| Poverty rate |  |  |  |  |  |  |
| Gross domestic product |  |  |  |  |  |  |
| Access to healthcare |  |  |  |  |  |  |
| Proportion of people of color |  |  |  |  |  |  |
| PM_2.5_ |  |  |  |  |  |  |
| Urban heat island intensity |  |  |  |  |  |  |
| Tree cover |  |  |  |  |  |  |
| Shrub-grass cover |  |  |  |  |  |  |
| Tree equity score |  |  |  |  |  |  |
| Health outcomes |  |  |  |  |  |  |
| Number of datasets | 9 | 9 | 10 | 10 | 9 | 8 |

**Table S3.** Distance-based PERMANOVA comparisons among city groups classified by poverty rate (HP, MP, and LP) and UMAP-based clustering (Groups A, B, and C). Pseudo-F values indicate the degree of multivariate separation between groups, while R² values represent the proportion of variation explained by group differences. Adjusted P-values were calculated using permutation tests with multiple-comparison correction. Significant differences were detected in all pairwise comparisons (adjusted P < 0.001), indicating that both classification approaches produced statistically distinguishable city groups with distinct multivariate characteristics. Abbreviations: HP, high poverty; MP, moderate poverty; LP, low poverty.

| Compare 1 | Compare 2 | Pseudo-F | R squared | Adjust P |
| --- | --- | --- | --- | --- |
| *City groups identified by poverty rate* | | |  |  |
| HP | LP | 93.22 | 0.22 | < 0.001 |
| HP | MP | 33.96 | 0.09 | < 0.001 |
| MP | LP | 17.41 | 0.05 | < 0.001 |
| *City groups identified by UMAP* | | |  |  |
| A | B | 48.79 | 0.13 | < 0.001 |
| A | C | 45.1 | 0.14 | < 0.001 |
| B | C | 82.25 | 0.17 | < 0.001 |

Table S4. Relative importance and rankings of explanatory variable categories for 11 health outcomes across U.S. city groups identified using Uniform Manifold Approximation and Projection (UMAP). Values represent the relative importance (%) of five explanatory variable categories: climate factors, socioeconomic conditions, racial composition, green infrastructure, and environmental hazards. Numbers in parentheses for each health outcome indicate the rank of the corresponding explanatory variable category based on its relative importance. Rank column represents the mean rank across the 11 health outcomes, and the values in parentheses indicate the standard deviation of ranks. Abbreviations: Climate, climate factors; Soc_econ, socioeconomic factors; Racial_com, racial composition; Env_haz, environmental hazard factors; Green_infra, green infrastructure factors; HP, high poverty; MP, moderate poverty; LP, low poverty; STD, standard deviation; HBP, high blood pressure; CHD, coronary heart disease; COPD, chronic obstructive pulmonary disease; HIGHCHOL, high cholesterol; CKD, chronic kidney disease; FMD, frequent mental distress.

| Factors | ARTHRITIS | HBP | ASTHMA | CHD | COPD | DIABETES | HIGHCHOL | CKD | FMD | OBESITY | STROKE | Rank |  |
| --- | --- | --- | --- | --- | --- | --- | --- | --- | --- | --- | --- | --- | --- |
|  |  |  |  |  |  |  |  |  |  |  |  |  |  |
| ***Group A*** |  |  |  |  |  |  |  |  |  |  |  |  |  |
| Climate | 12.85 (1) | 33.11 (1) | 0.43 (5) | 3.86 (3) | 1.59 (5) | 0.62 (5) | 0 (5) | 7.63 (3) | 13.6 (2) | 0.46 (5) | 14.95 (1) | 3.27(1.79) |  |
| Soc_econ | 0.86 (5) | 14.00 (2) | 4.17 (3) | 5.51 (2) | 3.27 (4) | 0.98 (4) | 9.16 (3) | 42.39 (1) | 7.75 (4) | 9.64 (3) | 0.17 (5) | 3.27(1.27) |  |
| Racial_com | 7.72 (3) | 11.20 (3) | 3.73 (4) | 0.13 (5) | 18.32 (2) | 7.14 (2) | 8.78 (4) | 0.65 (4) | 22.37 (1) | 10.61 (2) | 2.23 (3) | 3.00(1.18) |  |
| Env_haz | 5.30 (4) | 1.48 (5) | 5.45 (2) | 0.92 (4) | 11.51 (3) | 5.65 (3) | 9.17 (2) | 0 (5) | 4.80 (5) | 7.24 (4) | 1.20 (4) | 3.73(1.10) |  |
| Green_infra | 10.26 (2) | 5.68 (4) | 13.84 (1) | 11.79 (1) | 20.52 (1) | 7.79 (1) | 19.00 (1) | 27.72 (2) | 10.89 (3) | 15.58 (1) | 8.15 (2) | 1.73(1.01) |  |
| Residuals | 63.01 | 34.53 | 72.38 | 77.79 | 44.79 | 77.82 | 53.89 | 21.61 | 40.59 | 56.48 | 73.30 |  |  |
|  |  |  |  |  |  |  |  |  |  |  |  |  |  |
| ***Group B*** |  |  |  |  |  |  |  |  |  |  |  |  |  |
| Climate | 0.07 (5) | 5.27 (3) | 0.56 (5) | 0.11 (5) | 5.11 (3) | 1.10 (5) | 2.19 (4) | 0.54 (5) | 8.08 (3) | 0.50 (4) | 0 (5) | 4.27(0.90) |  |
| Soc_econ | 4.22 (2) | 3.77 (4) | 36.99 (1) | 5.61 (2) | 10.60 (2) | 4.50 (4) | 6.09 (2) | 12.91 (2) | 42.15 (1) | 17.34 (1) | 10.25 (2) | 2.09(1.04) |  |
| Racial_com | 0.07 (4) | 6.06 (2) | 7.76 (3) | 0.13 (4) | 0 (5) | 14.93 (2) | 0 (5) | 8.26 (3) | 4.03 (4) | 11.87 (3) | 6.87 (3) | 3.45(1.04) |  |
| Env_haz | 0.44 (3) | 1.13 (5) | 1.00 (4) | 2.96 (3) | 4.08 (4) | 4.90 (3) | 2.45 (3) | 4.35 (4) | 1.02 (5) | 0.23 (5) | 4.81 (4) | 3.91(0.83) |  |
| Green_infra | 8.85 (1) | 8.99 (1) | 8.07 (2) | 15.23 (1) | 14.85 (1) | 21.50 (1) | 7.44 (1) | 17.91 (1) | 23.53 (2) | 15.2 (2) | 18.2 (1) | 1.27(0.47) |  |
| Residuals | 86.35 | 74.78 | 45.62 | 75.96 | 65.36 | 53.07 | 81.83 | 56.03 | 21.19 | 54.86 | 59.87 |  |  |
|  |  |  |  |  |  |  |  |  |  |  |  |  |  |
| ***Group C*** |  |  |  |  |  |  |  |  |  |  |  |  |  |
| Climate | 3.87 (4) | 0.72 (4) | 0 (5) | 1.00 (5) | 1.34 (4) | 6.54 (4) | 3.34 (3) | 1.37 (4) | 5.86 (4) | 9.88 (3) | 0.16 (3) | 3.91(0.70) |  |
| Soc_econ | 6.65 (3) | 8.98 (1) | 24.92 (1) | 12.28 (1) | 20.20 (1) | 15.67 (2) | 0.85 (5) | 22.49 (1) | 34.49 (1) | 14.75 (1) | 23.84 (1) | 1.64(1.29) |  |
| Racial_com | 10.62 (1) | 0.21 (5) | 5.31 (3) | 2.46 (3) | 6.94 (3) | 19.01 (1) | 2.53 (4) | 5.68 (3) | 0.67 (5) | 3.20 (4) | 0 (5) | 3.36(1.43) |  |
| Env_haz | 0 (5) | 0.89 (3) | 0.06 (4) | 1.80 (4) | 0.51 (5) | 4.20 (5) | 3.42 (2) | 0 (5) | 5.86 (3) | 0 (5) | 0.13 (4) | 4.09(1.04) |  |
| Green_infra | 7.83 (2) | 5.99 (2) | 11.00 (2) | 7.74 (2) | 9.66 (2) | 8.03 (3) | 4.05 (1) | 9.98 (2) | 19.87 (2) | 10.81 (2) | 10.24 (2) | 2.00(0.45) |  |
| Residuals | 71.03 | 83.21 | 58.71 | 74.72 | 61.35 | 46.55 | 85.81 | 60.48 | 33.25 | 61.36 | 65.63 |  |  |

**Table S5.** Effects of tree equity score (TES) on the prevalence of 11 health outcomes and projected health gains under hypothetical TES improvements for all U.S. cities, poverty rate-based city groups (Group HP, Group MP, Group LP), and UMAP-identified city groups (Group A, Group B, and Group C). For the health impact and sensitivity analyses (ΔTES = 1, 3, and 5) presented in this table, we refitted the models using unstandardized coefficients and used the unstandardized total effect of TES on each health outcome to derive absolute changes in prevalence and changes in cases per 100,000 residents for ΔTES = 5. Abbreviations for the health outcomes are shown in Table S4.

| Group | Health outcomes |  | TES effect^1^ | | |  | Baseline (%)^2^ |  | Prevalence^3^ | | |  | ΔCase_5  per 100,000^4^ |
| --- | --- | --- | --- | --- | --- | --- | --- | --- | --- | --- | --- | --- | --- |
|  |  |  | Direct | Indirect | Total |  |  |  | ΔTES_1 | ΔTES_3 | ΔTES_5 |  |  |
| All cities | ARTHRITIS |  | –0.1775 | 0 | –0.1775 |  | 21.15 |  | 20.97±0.05 | 20.61±0.14 | 20.26±0.23 |  | 887.5±235.0 |
|  |  |  |  |  |  |  |  |  | (0.84±0.22) | (2.52±0.67) | (4.20±1.11) |  |  |
|  | HBP |  | –0.1611 | 0 | –0.1611 |  | 29.58 |  | 29.42±0.05 | 29.10±0.14 | 28.78±0.23 |  | 805.5±226.5 |
|  |  |  |  |  |  |  |  |  | (0.54±0.15) | (1.63±0.46) | (2.72±0.77) |  |  |
|  | ASTHMA |  | –0.1248 | 0 | –0.1248 |  | 9.63 |  | 9.51±0.03 | 9.26±0.10 | 9.01±0.17 |  | 624±165.5 |
|  |  |  |  |  |  |  |  |  | (1.30±0.34) | (3.89±1.03) | (6.48±1.72) |  |  |
|  | CHD |  | –0.2577 | 0 | –0.2577 |  | 5.42 |  | 5.17±0.04 | 4.65±0.13 | 4.14±0.22 |  | 1288.5±218.5 |
|  |  |  |  |  |  |  |  |  | (4.75±0.81) | (14.25±2.42) | (23.75±4.03) |  |  |
|  | COPD |  | –0.2680 | 0 | –0.2680 |  | 6.07 |  | 5.81±0.04 | 5.27±0.11 | 4.73±0.18 |  | 1340±184.5 |
|  |  |  |  |  |  |  |  |  | (4.41±0.61) | (13.24±1.82) | (22.06±3.04) |  |  |
|  | DIABETES |  | –0.2094 | 0 | –0.2094 |  | 10.51 |  | 10.30±0.04 | 9.88±0.12 | 9.46±0.20 |  | 1047±203.0 |
|  |  |  |  |  |  |  |  |  | (1.99±0.39) | (5.98±1.16) | (9.96±1.93) |  |  |
|  | HIGHCHOL |  | –0.1661 | 0 | –0.1661 |  | 30.57 |  | 30.41±0.05 | 30.07±0.15 | 29.74±0.25 |  | 830.5±249.5 |
|  |  |  |  |  |  |  |  |  | (0.54±0.16) | (1.63±0.49) | (2.72±0.82) |  |  |
|  | CKD |  | –0.2050 | 0 | –0.2050 |  | 2.90 |  | 2.70±0.04 | 2.29±0.14 | 1.88±0.22 |  | 1025±225.0 |
|  |  |  |  |  |  |  |  |  | (7.07±1.55) | (21.20±4.65) | (35.34±7.76) |  |  |
|  | FMD |  | –0.2810 | 0 | –0.2810 |  | 14.31 |  | 14.02±0.03 | 13.46±0.09 | 12.90±0.15 |  | 1405.0±147.0 |
|  |  |  |  |  |  |  |  |  | (1.96±0.21) | (5.89±0.62) | (9.82±1.03) |  |  |
|  | OBESITY |  | –0.2221 | 0 | –0.2221 |  | 30.27 |  | 30.05±0.04 | 29.61±0.11 | 29.16±0.18 |  | 1110.5±181.0 |
|  |  |  |  |  |  |  |  |  | (0.73±0.12) | (2.20±0.36) | (3.67±0.60) |  |  |
|  | STROKE |  | –0.2006 | 0 | –0.2006 |  | 3.10 |  | 2.90±0.05 | 2.50±0.14 | 2.09±0.23 |  | 1003.0±226.5 |
|  |  |  |  |  |  |  |  |  | (6.48±1.46) | (19.43±4.39) | (32.38±7.31) |  |  |
| *City groups classified by poverty rate* | | | |  |  |  |  |  |  |  |  |  |  |
|  |  |  |  |  |  |  |  |  |  |  |  |  |  |
| Group HP | ARTHRITIS |  | –0.4424 | 0 | –0.4424 |  | 22.91 |  | 22.47±0.08 | 21.58±0.23 | 20.70±0.38 |  | 2212±379.0 |
|  |  |  |  |  |  |  |  |  | (1.93±0.33) | (5.79±0.99) | (9.66±1.65) |  |  |
|  | HBP |  | –0.3270 | 0 | –0.3270 |  | 32.20 |  | 31.88±0.07 | 31.22±0.21 | 30.57±0.35 |  | 1635±352.5 |
|  |  |  |  |  |  |  |  |  | (1.02±0.22) | (3.05±0.66) | (5.08±1.09) |  |  |
|  | ASTHMA |  | –0.1540 | 0 | –0.1540 |  | 10.41 |  | 10.25±0.06 | 9.94±0.18 | 9.64±0.29 |  | 770±292.0 |
|  |  |  |  |  |  |  |  |  | (1.48±0.56) | (4.44±1.68) | (7.40±2.81) |  |  |
|  | CHD |  | –0.4805 | 0 | –0.4805 |  | 6.03 |  | 5.55±0.08 | 4.59±0.23 | 3.63±0.38 |  | 2402.5±375.5 |
|  |  |  |  |  |  |  |  |  | (7.97±1.25) | (23.91±3.74) | (39.86±6.23) |  |  |
|  | COPD |  | –0.4017 | 0 | –0.4017 |  | 7.00 |  | 6.60±0.07 | 5.80±0.21 | 4.99±0.35 |  | 2008.5±352.0 |
|  |  |  |  |  |  |  |  |  | (5.74±1.01) | (17.21±3.02) | (28.68±5.03) |  |  |
|  | DIABETES |  | –0.2793 | 0 | –0.2793 |  | 11.84 |  | 11.56±0.06 | 11.00±0.18 | 10.44±0.30 |  | 1396.5±304.0 |
|  |  |  |  |  |  |  |  |  | (2.36±0.51) | (7.08±1.54) | (11.80±2.57) |  |  |
|  | HIGHCHOL |  | –0.4066 | 0 | –0.4066 |  | 30.70 |  | 30.30±0.07 | 29.48±0.22 | 28.67±0.37 |  | 2033±370.5 |
|  |  |  |  |  |  |  |  |  | (1.32±0.24) | (3.97±0.72) | (6.62±1.21) |  |  |
|  | CKD |  | –0.3388 | 0 | –0.3388 |  | 3.19 |  | 2.85±0.07 | 2.18±0.20 | 1.50±0.34 |  | 1694±339.5 |
|  |  |  |  |  |  |  |  |  | (10.61±2.13) | (31.84±6.38) | (53.06±10.63) |  |  |
|  | FMD |  | –0.2326 | 0 | –0.2326 |  | 15.99 |  | 15.72±0.06 | 15.18±0.17 | 14.65±0.28 |  | 1338.8±278.0 |
|  |  |  |  |  |  |  |  |  | (1.67±0.35) | (5.02±1.04) | (8.37±1.74) |  |  |
|  | OBESITY |  | –0.3109 | 0 | –0.3109 |  | 34.13 |  | 33.81±0.06 | 33.19±0.19 | 32.57±0.32 |  | 1554.5±315.0 |
|  |  |  |  |  |  |  |  |  | (0.91±0.18) | (2.73±0.55) | (4.56±0.92) |  |  |
|  | STROKE |  | –0.3112 | 0 | –0.3112 |  | 3.58 |  | 3.26±0.07 | 2.64±0.21 | 2.02±0.35 |  | 1556.0±355.0 |
|  |  |  |  |  |  |  |  |  | (8.70±1.99) | (26.11±5.96) | (43.52±9.93) |  |  |
| Group MP | ARTHRITIS |  | 0 | 0 | 0 |  | 20.45 |  | 20.45±0.00 | 20.45±0.00 | 20.45±0.00 |  | 0 |
|  |  |  |  |  |  |  |  |  | (0.00±0.00) | (0.00±0.00) | (0.00±0.00) |  |  |
|  | HBP |  | –0.1441 | 0 | –0.1441 |  | 29.02 |  | 28.87±0.07 | 28.58±0.21 | 28.29±0.35 |  | 720.5±348.0 |
|  |  |  |  |  |  |  |  |  | (0.50±0.24) | (1.49±0.72) | (2.48±1.20) |  |  |
|  | ASTHMA |  | 0 | 0 | 0 |  | 9.50 |  | 9.50±0.00 | 9.50±0.00 | 9.50±0.00 |  | 0 |
|  |  |  |  |  |  |  |  |  | (0.00±0.00) | (0.00±0.00) | (0.00±0.00) |  |  |
|  | CHD |  | 0 | 0 | 0 |  | 5.35 |  | 5.35±0.00 | 5.35±0.00 | 5.35±0.00 |  | 0 |
|  |  |  |  |  |  |  |  |  | (0.00±0.00) | (0.00±0.00) | (0.00±0.00) |  |  |
|  | COPD |  | –0.1676 | 0 | –0.1676 |  | 5.92 |  | 5.76±0.07 | 5.42±0.20 | 5.09±0.33 |  | 838±331.5 |
|  |  |  |  |  |  |  |  |  | (2.83±1.12) | (8.49±3.36) | (14.15±5.60) |  |  |
|  | DIABETES |  | –0.1674 | 0 | –0.1674 |  | 10.40 |  | 10.23±0.07 | 9.90±0.21 | 9.56±0.36 |  | 837±358.0 |
|  |  |  |  |  |  |  |  |  | (1.61±0.69) | (4.83±2.07) | (8.05±3.44) |  |  |
|  | HIGHCHOL |  | 0 | 0 | 0 |  | 30.64 |  | 30.64±0.00 | 30.64±0.00 | 30.64±0.00 |  | 0 |
|  |  |  |  |  |  |  |  |  | (0.00±0.00) | (0.00±0.00) | (0.00±0.00) |  |  |
|  | CKD |  | 0 | 0 | 0 |  | 2.88 |  | 2.88±0.00 | 2.88±0.00 | 2.88±0.00 |  | 0 |
|  |  |  |  |  |  |  |  |  | (0.00±0.00) | (0.00±0.00) | (0.00±0.00) |  |  |
|  | FMD |  | –0.2609 | 0 | –0.2609 |  | 14.08 |  | 13.82±0.08 | 13.30±0.24 | 12.78±0.40 |  | 1304.5±404.0 |
|  |  |  |  |  |  |  |  |  | (1.85±0.57) | (5.56±1.72) | (9.26±2.87) |  |  |
|  | OBESITY |  | –0.2740 | 0 | –0.2740 |  | 29.43 |  | 29.16±0.07 | 28.61±0.22 | 28.06±0.37 |  | 1370±368.0 |
|  |  |  |  |  |  |  |  |  | (0.93±0.25) | (2.79±0.75) | (4.65±1.25) |  |  |
|  | STROKE |  | 0 | 0 | 0 |  | 3.03 |  | 3.03±0.00 | 3.03±0.00 | 3.03±0.00 |  | 0 |
|  |  |  |  |  |  |  |  |  | (0.00±0.00) | (0.00±0.00) | (0.00±0.00) |  |  |
| Group LP | ARTHRITIS |  | –0.2012 | 0 | –0.2012 |  | 19.97 |  | 19.77±0.08 | 19.36±0.23 | 18.96±0.39 |  | 1006±386.0 |
|  |  |  |  |  |  |  |  |  | (1.01±0.39) | (3.02±1.16) | (5.04±1.93) |  |  |
|  | HBP |  | –0.2472 | 0 | –0.2472 |  | 26.77 |  | 26.52±0.09 | 26.03±0.27 | 25.53±0.45 |  | 1236±447.5 |
|  |  |  |  |  |  |  |  |  | (0.92±0.33) | (2.77±1.00) | (4.62±1.67) |  |  |
|  | ASTHMA |  | –0.2418 | 0 | –0.2418 |  | 8.74 |  | 8.50±0.07 | 8.02±0.21 | 7.53±0.35 |  | 1209.0±345.5 |
|  |  |  |  |  |  |  |  |  | (2.77±0.79) | (8.30±2.37) | (13.83±3.95) |  |  |
|  | CHD |  | –0.3164 | 0 | –0.3164 |  | 4.64 |  | 4.33±0.09 | 3.69±0.27 | 3.06±0.44 |  | 1582±445.0 |
|  |  |  |  |  |  |  |  |  | (6.81±1.92) | (20.44±5.75) | (34.07±9.58) |  |  |
|  | COPD |  | –0.4077 | 0 | –0.4077 |  | 4.95 |  | 4.55±0.07 | 3.73±0.21 | 2.92±0.35 |  | 2038.5±352.0 |
|  |  |  |  |  |  |  |  |  | (8.23±1.42) | (24.68±4.26) | (41.14±7.10) |  |  |
|  | DIABETES |  | –0.3319 | 0 | –0.3319 |  | 8.69 |  | 8.36±0.08 | 7.69±0.24 | 7.03±0.40 |  | 1659.5±402.0 |
|  |  |  |  |  |  |  |  |  | (3.82±0.93) | (11.46±2.78) | (19.10±4.63) |  |  |
|  | HIGHCHOL |  | 0 | 0 | 0 |  | 30.22 |  | 30.22±0.00 | 30.22±0.00 | 30.22±0.00 |  | 0 |
|  |  |  |  |  |  |  |  |  | (0.00±0.00) | (0.00±0.00) | (0.00±0.00) |  |  |
|  | CKD |  | 0 | 0 | 0 |  | 2.49 |  | 2.49±0.00 | 2.49±0.00 | 2.49±0.00 |  | 0 |
|  |  |  |  |  |  |  |  |  | (0.00±0.00) | (0.00±0.00) | (0.00±0.00) |  |  |
|  | FMD |  | –0.4432 | 0 | –0.4432 |  | 12.17 |  | 11.73±0.07 | 10.84±0.20 | 9.96±0.33 |  | 2216.0±326.5 |
|  |  |  |  |  |  |  |  |  | (3.64±0.54) | (10.92±1.61) | (18.21±2.68) |  |  |
|  | OBESITY |  | –0.2857 | 0 | –0.2857 |  | 26.13 |  | 25.85±0.07 | 25.28±0.20 | 24.70±0.33 |  | 1428.5±327.5 |
|  |  |  |  |  |  |  |  |  | (1.09±0.25) | (3.28±0.75) | (5.47±1.25) |  |  |
|  | STROKE |  | –0.3462 | 0 | –0.3462 |  | 2.51 |  | 2.16±0.09 | 1.47±0.26 | 0.78±0.43 |  | 1731±434.5 |
|  |  |  |  |  |  |  |  |  | (13.79±3.46) | (41.38±10.39) | (68.96±17.31) |  |  |
| *City groups identified by UMAP* | | | |  |  |  |  |  |  |  |  |  |  |
|  |  |  |  |  |  |  |  |  |  |  |  |  |  |
| Group A | ARTHRITIS |  | –0.4421 | 0 | –0.4421 |  | 23.21 |  | 22.76±0.10 | 21.88±0.29 | 21.00±0.49 |  | 2210.5±488.5 |
|  |  |  |  |  |  |  |  |  | (1.91±0.42) | (5.72±1.26) | (9.53±2.11) |  |  |
|  | HBP |  | –0.4828 | 0 | –0.4828 |  | 33.57 |  | 33.09±0.12 | 32.12±0.35 | 31.15±0.58 |  | 2414±582.0 |
|  |  |  |  |  |  |  |  |  | (1.44±0.35) | (4.31±1.04) | (7.19±1.73) |  |  |
|  | ASTHMA |  | 0 | 0 | 0 |  | 9.65 |  | 9.65±0.00 | 9.65±0.00 | 9.65±0.00 |  | 0 |
|  |  |  |  |  |  |  |  |  | (0.00±0.00) | (0.00±0.00) | (0.00±0.00) |  |  |
|  | CHD |  | –0.5597 | –0.0867 | –0.6464 |  | 6.10 |  | 5.46±0.12 | 4.16±0.35 | 2.87±0.58 |  | 3232.2±582.5 |
|  |  |  |  |  |  |  |  |  | (10.59±1.91) | (31.78±5.73) | (52.96±9.54) |  |  |
|  | COPD |  | –0.5232 | 0 | –0.5232 |  | 7.13 |  | 6.61±0.11 | 5.56±0.32 | 4.52±0.53 |  | 2616±527.0 |
|  |  |  |  |  |  |  |  |  | (7.33±1.48) | (22.00±4.43) | (36.67±7.39) |  |  |
|  | DIABETES |  | –0.4513 | –0.1080 | –0.5593 |  | 11.70 |  | 11.14±0.10 | 10.03±0.31 | 8.91±0.51 |  | 2796.7±510.0 |
|  |  |  |  |  |  |  |  |  | (4.78±0.87) | (14.34±2.61) | (23.90±4.36) |  |  |
|  | HIGHCHOL |  | –0.3722 | –0.1031 | –0.4753 |  | 32.16 |  | 31.69±0.13 | 30.74±0.39 | 29.79±0.64 |  | 2376.7±642.0 |
|  |  |  |  |  |  |  |  |  | (1.48±0.40) | (4.43±1.20) | (7.39±2.00) |  |  |
|  | CKD |  | –0.4876 | –0.0945 | –0.5821 |  | 3.11 |  | 2.52±0.10 | 1.36±0.31 | 0.20±0.52 |  | 2910.6±516.0 |
|  |  |  |  |  |  |  |  |  | (18.74±3.32) | (56.22±9.97) | (93.70±16.61) |  |  |
|  | FMD |  | –0.2901 | 0 | –0.2901 |  | 15.20 |  | 14.91±0.06 | 14.33±0.19 | 13.75±0.31 |  | 1450.5±310.5 |
|  |  |  |  |  |  |  |  |  | (1.91±0.41) | (5.73±1.23) | (9.54±2.04) |  |  |
|  | OBESITY |  | –0.2591 | 0 | –0.2591 |  | 33.43 |  | 33.17±0.09 | 32.65±0.26 | 32.14±0.44 |  | 1295.5±440.5 |
|  |  |  |  |  |  |  |  |  | (0.78±0.26) | (2.33±0.79) | (3.88±1.32) |  |  |
|  | STROKE |  | –0.4075 | –0.0782 | –0.4857 |  | 3.54 |  | 3.06±0.11 | 2.08±0.33 | 1.11±0.54 |  | 2428.6±544.0 |
|  |  |  |  |  |  |  |  |  | (13.72±3.07) | (41.15±9.22) | (68.58±15.36) |  |  |
| Group B | ARTHRITIS |  | –0.2388 | 0 | –0.2388 |  | 22.10 |  | 21.86±0.06 | 21.38±0.18 | 20.90±0.30 |  | 1194±296.5 |
|  |  |  |  |  |  |  |  |  | (1.08±0.27) | (3.24±0.81) | (5.40±1.34) |  |  |
|  | HBP |  | –0.2258 | 0 | –0.2258 |  | 29.45 |  | 29.23±0.06 | 28.78±0.17 | 28.33±0.28 |  | 1129±277.5 |
|  |  |  |  |  |  |  |  |  | (0.77±0.19) | (2.30±0.57) | (3.83±0.94) |  |  |
|  | ASTHMA |  | –0.1208 | 0 | –0.1208 |  | 10.22 |  | 10.10±0.04 | 9.85±0.13 | 9.61±0.22 |  | 604±218.5 |
|  |  |  |  |  |  |  |  |  | (1.18±0.43) | (3.55±1.28) | (5.91±2.14) |  |  |
|  | CHD |  | –0.3186 | –0.0088 | –0.3274 |  | 5.50 |  | 5.18±0.06 | 4.52±0.18 | 3.87±0.30 |  | 1636.8±305.0 |
|  |  |  |  |  |  |  |  |  | (5.95±1.11) | (17.84±3.32) | (29.74±5.54) |  |  |
|  | COPD |  | –0.3448 | 0 | –0.3448 |  | 6.29 |  | 5.95±0.05 | 5.26±0.16 | 4.57±0.27 |  | 1724±266.5 |
|  |  |  |  |  |  |  |  |  | (5.48±0.85) | (16.44±2.54) | (27.40±4.24) |  |  |
|  | DIABETES |  | –0.2896 | –0.0088 | –0.2984 |  | 10.29 |  | 9.99±0.05 | 9.39±0.15 | 8.80±0.25 |  | 1492.2±250.5 |
|  |  |  |  |  |  |  |  |  | (2.90±0.49) | (8.70±1.46) | (14.50±2.43) |  |  |
|  | HIGHCHOL |  | –0.2770 | 0 | –0.2770 |  | 30.11 |  | 29.84±0.07 | 29.28±0.20 | 28.73±0.34 |  | 1385.0±337.5 |
|  |  |  |  |  |  |  |  |  | (0.92±0.22) | (2.76±0.67) | (4.60±1.12) |  |  |
|  | CKD |  | –0.2409 | –0.0074 | –0.2483 |  | 2.88 |  | 2.63±0.06 | 2.14±0.17 | 1.64±0.28 |  | 1241.3±283.5 |
|  |  |  |  |  |  |  |  |  | (8.62±1.97) | (25.85±5.90) | (43.09±9.84) |  |  |
|  | FMD |  | –0.2423 | 0 | –0.2423 |  | 14.50 |  | 14.26±0.03 | 13.78±0.10 | 13.29±0.16 |  | 1211.5±162.5 |
|  |  |  |  |  |  |  |  |  | (1.67±0.22) | (5.01±0.67) | (8.35±1.12) |  |  |
|  | OBESITY |  | –0.2693 | 0 | –0.2693 |  | 30.18 |  | 29.91±0.05 | 29.37±0.15 | 28.83±0.25 |  | 1346.5±252.5 |
|  |  |  |  |  |  |  |  |  | (0.89±0.17) | (2.68±0.50) | (4.46±0.84) |  |  |
|  | STROKE |  | –0.2771 | –0.0078 | –0.2849 |  | 3.16 |  | 2.88±0.06 | 2.31±0.17 | 1.74±0.29 |  | 1424.4±290.5 |
|  |  |  |  |  |  |  |  |  | (9.01±1.84) | (27.02±5.51) | (45.03±9.18) |  |  |
| Group C | ARTHRITIS |  | –0.3198 | 0 | –0.3198 |  | 18.99 |  | 18.67±0.08 | 18.03±0.23 | 17.39±0.39 |  | 1599.0±389.5 |
|  |  |  |  |  |  |  |  |  | (1.68±0.41) | (5.05±1.23) | (8.42±2.05) |  |  |
|  | HBP |  | –0.2333 | 0 | –0.2333 |  | 27.61 |  | 27.38±0.07 | 26.91±0.21 | 26.44±0.35 |  | 1166.5±348.5 |
|  |  |  |  |  |  |  |  |  | (0.84±0.25) | (2.53±0.76) | (4.22±1.26) |  |  |
|  | ASTHMA |  | –0.3132 | 0 | –0.3132 |  | 8.98 |  | 8.67±0.06 | 8.04±0.19 | 7.41±0.32 |  | 1566.0±324.5 |
|  |  |  |  |  |  |  |  |  | (3.49±0.72) | (10.46±2.17) | (17.44±3.61) |  |  |
|  | CHD |  | –0.2999 | 0 | –0.2999 |  | 4.97 |  | 4.67±0.08 | 4.07±0.23 | 3.47±0.39 |  | 1499.5±386.5 |
|  |  |  |  |  |  |  |  |  | (6.03±1.55) | (18.09±4.66) | (30.15±7.77) |  |  |
|  | COPD |  | –0.4011 | 0 | –0.4011 |  | 5.27 |  | 4.87±0.07 | 4.06±0.20 | 3.26±0.34 |  | 2005.5±339.0 |
|  |  |  |  |  |  |  |  |  | (7.62±1.29) | (22.85±3.86) | (38.08±6.44) |  |  |
|  | DIABETES |  | –0.1980 | 0 | –0.1980 |  | 10.12 |  | 9.93±0.06 | 9.53±0.19 | 9.13±0.31 |  | 990±314.0 |
|  |  |  |  |  |  |  |  |  | (1.96±0.62) | (5.87±1.86) | (9.78±3.10) |  |  |
|  | HIGHCHOL |  | –0.2502 | 0.0289 | –0.2213 |  | 30.24 |  | 30.01±0.09 | 29.57±0.27 | 29.13±0.45 |  | 1106.4±452.0 |
|  |  |  |  |  |  |  |  |  | (0.73±0.30) | (2.20±0.90) | (3.66±1.49) |  |  |
|  | CKD |  | 0 | 0 | 0 |  | 2.81 |  | 2.81±0.00 | 2.81±0.00 | 2.81±0.00 |  | 0 |
|  |  |  |  |  |  |  |  |  | (0.00±0.00) | (0.00±0.00) | (0.00±0.00) |  |  |
|  | FMD |  | –0.3622 | 0.0182 | –0.3440 |  | 13.61 |  | 13.27±0.05 | 12.58±0.14 | 11.89±0.23 |  | 1719.9±232.0 |
|  |  |  |  |  |  |  |  |  | (2.53±0.34) | (7.58±1.02) | (12.64±1.70) |  |  |
|  | OBESITY |  | –0.2222 | 0 | –0.2222 |  | 28.70 |  | 28.47±0.06 | 28.03±0.17 | 27.58±0.28 |  | 1111±278.0 |
|  |  |  |  |  |  |  |  |  | (0.77±0.19) | (2.32±0.58) | (3.87±0.97) |  |  |
|  | STROKE |  | –0.2858 | 0 | –0.2858 |  | 2.79 |  | 2.50±0.06 | 1.93±0.19 | 1.36±0.32 |  | 1429±319.5 |
|  |  |  |  |  |  |  |  |  | (10.25±2.29) | (30.74±6.87) | (51.23±11.45) |  |  |

^1^ TES effect refers to direct, indirect, and total effects of TES on health outcome prevalence derived from piecewise structural equation models (see Figures S14-S27). ‘Direct’ is the unstandardized path coefficient for the direct effect of TES on disease prevalence, ‘Indirect’ is the sum of all indirect effects operating through intermediate variables (e.g., urban heat island intensity, PM_2.5_), and ‘Total’ is the sum of direct and indirect effects.

^2^ Baseline (%) is the population-weighted mean prevalence of each disease.

^3^ Prevalence values represent the model-predicted prevalence (%) under scenarios in which TES increases by 1 point (ΔTES_1), 3 points (ΔTES_3), or 5 points (ΔTES_5). Values are presented as mean ± standard deviation (SD). Values in parentheses indicate the percentage reduction relative to the baseline prevalence and are also presented as mean ± SD.

^4^ ΔCases_5 per 100,000 denotes the predicted reduction in the number of cases per 100,000 residents associated with a 5-point increase in TES. Values are presented as mean ± SD

Fig. S1. A map showing the geographical locations of U.S. cities and the prevalence of 11 health outcomes in each city. Abbreviations: HBP, blood pressure high; CHD, coronary heart disease; COPD, chronic obstructive pulmonary disease; HIGHCHOL, high cholesterol; CKD, chronic kidney disease; FMD, frequent mental distress.

Fig. S2. Distribution of the 497 U.S. cities and variation in explanatory variables and health outcome prevalence across city groups identified using Uniform Manifold Approximation and Projection (UMAP): (a) spatial distribution of cities, (b) UMAP-based clustering of cities, (c) distributions of explanatory variables among UMAP-defined city groups, and (d) distributions of health outcome prevalence among UMAP-defined city groups. Abbreviations: MAT, mean annual temperature; MAP, mean annual precipitation; GDP_capita, gross domestic product per capita; POV, poverty rate; AH, access to healthcare; POC, proportion of people of color; UHI, urban heat-island intensity; PM2.5, fine particulate matter with an aerodynamic diameter of no more than 2.5 μm; Tree, tree cover; S_G, shrub-grass cover; TES, tree equity score; HBP, high blood pressure; CHD, coronary heart disease; COPD, chronic obstructive pulmonary disease; HIGHCHOL, high cholesterol; CKD, chronic kidney disease; FMD, frequent mental distress.

Fig. S3. Distribution of the 497 U.S. cities and variation in explanatory variables and health outcome prevalence across city groups identified using Uniform Manifold Approximation and Projection (UMAP): (a) spatial distribution of cities assigned to UMAP-defined groups, (b) UMAP-based clustering of cities, (c) distributions of explanatory variables among UMAP-defined city groups, and (d) distributions of health outcome prevalence among UMAP-defined city groups. Spatial distributions of the six clusters identified by UMAP are shown in Fig. S2. Abbreviations are provided in Fig. S2.

**Fig. S4.** Pearson correlation coefficients among control factors (a) total cities, (b) city groups classified by poverty level, and (c) city groups identified by Uniform Manifold Approximation and Projection (UMAP) in the United States. Abbreviations: POP_Den, population density; GS, green space cover; NDVI, normalized difference vegetation index; log, log transformation; SQRT, square-root transformation. Abbreviations for the remaining variables are shown in Fig. S2.

Fig. S5. A conceptual model illustrating how climate, socioeconomic conditions, racial composition, green infrastructure, and environmental hazards that determine the prevalence of 11 health outcomes in the United States. Socioeconomic factors comprise economic components (i.e., poverty and GDP per capita) and a social component (i.e., access to healthcare). Based on this model, we tested causal relationships among factors and evaluate their direct and indirect effects on the 11 health outcomes. Abbreviations are shown in Fig. S2.

**Fig. S6.** Standardized parameter estimates of effect sizes (circles) and standard errors (bars) of each control factor on the prevalence of 11 health outcomes across all U.S. cities. Closed and open circles indicate significant (P < 0.05) and non-significant relationships, respectively. The relative importance of each factor was calculated as the ratio of the parameter estimate for that factor to the sum of parameter estimates across all factors, and then expressed as a percentage. Abbreviations for the variables are shown in Fig. S2.

**Fig. S7.** Standardized parameter estimates of effect sizes (circles) and standard errors (bars) of each control factor on the prevalence of 11 health outcomes in the high-poverty city group, one of the city groups classified by poverty level in the United States. Closed and open circles indicate significant (P < 0.05) and non-significant relationships, respectively. The relative importance of each factor was calculated as the ratio of the parameter estimate for that factor to the sum of parameter estimates across all factors, and then expressed as a percentage. Abbreviations for the variables are shown in Fig. S2.

**Fig. S8.** Standardized parameter estimates of effect sizes (circles) and standard errors (bars) of each control factor on the prevalence of 11 health outcomes in the moderate-poverty city group, one of the city groups classified by poverty level in the United States. Closed and open circles indicate significant (P < 0.05) and non-significant relationships, respectively. The relative importance of each factor was calculated as the ratio of the parameter estimate for that factor to the sum of parameter estimates across all factors, and then expressed as a percentage. Abbreviations for the variables are shown in Fig. S2.

Fig. S9. Standardized parameter estimates of effect sizes (circles) and standard errors (bars) of each control factor on the prevalence of 11 health outcomes in the low-poverty city group, one of the city groups classified by poverty level in the United States. Closed and open circles indicate significant (P < 0.05) and non-significant relationships, respectively. The relative importance of each factor was calculated as the ratio of the parameter estimate for that factor to the sum of parameter estimates across all factors, and then expressed as a percentage. Abbreviations for the variables are shown in Fig. S2.

**Fig. S10.** Standardized parameter estimates of effect sizes (circles) and standard errors (bars) of each control factor on the prevalence of 11 health outcomes in Group A, one of the city groups identified by Uniform Manifold Approximation and Projection (UMAP) in the United States. Closed and open circles indicate significant (P < 0.05) and non-significant relationships, respectively. The relative importance of each factor was calculated as the ratio of the parameter estimate for that factor to the sum of parameter estimates across all factors, and then expressed as a percentage. Abbreviations for the variables are shown in Fig. S2.

**Fig. S11.** Standardized parameter estimates of effect sizes (circles) and standard errors (bars) of each control factor on the prevalence of 11 health outcomes in Group B, one of the city groups identified by Uniform Manifold Approximation and Projection (UMAP) in the United States. Closed and open circles indicate significant (P < 0.05) and non-significant relationships, respectively. The relative importance of each factor was calculated as the ratio of the parameter estimate for that factor to the sum of parameter estimates across all factors, and then expressed as a percentage. Abbreviations for the variables are shown in Fig. S2.

**Fig. S12.** Standardized parameter estimates of effect sizes (circles) and standard errors (bars) of each control factor on the prevalence of 11 health outcomes in Group C, one of the city groups identified by Uniform Manifold Approximation and Projection (UMAP) in the United States. Closed and open circles indicate significant (P < 0.05) and non-significant relationships, respectively. The relative importance of each factor was calculated as the ratio of the parameter estimate for that factor to the sum of parameter estimates across all factors, and then expressed as a percentage. Abbreviations for the variables are shown in Fig. S2.

**Fig. S13.** The figure shows the causal relationships among climate, socioeconomic conditions, racial composition, green infrastructure, and environmental hazards for the prevalence of the five health outcomes with the highest coefficients of determination (R^2^), derived from piecewise structural equation modeling across all U.S. cities. Socioeconomic factors (Soc_econ in the figure) comprise economic components (poverty and GDP per capita) and a social component (access to healthcare). Abbreviations: Climate, climate factors; Green infra, green infrastructure factors; Env_hazard, environment hazard factors; Soc_econ, socioeconomic factors; Racial, racial composition. Abbreviations for the other variables are shown in Fig. S2.

**Fig. S14.** Direct and indirect associations among climate factors, socioeconomic conditions, racial composition, green infrastructure, environmental hazards, and the prevalence of the five health outcomes with the highest coefficients of determination (R²) across all U.S. cities, derived from piecewise structural equation modeling (pSEM). Socioeconomic factors (Soc_econ in the figure) comprise economic components (poverty rate and GDP per capita) and a social component (access to healthcare). Abbreviations: Climate, climate factors; Green infra, green infrastructure factors; Env_hazard, environmental hazard factors; Soc_econ, socioeconomic factors; Racial, racial composition. Abbreviations for the remaining variables are provided in Fig. S2.

Fig. S15. Direct and indirect associations among climate factors, socioeconomic conditions, racial composition, green infrastructure, environmental hazards, and the prevalence of the five health outcomes with the highest coefficients of determination (R²) in the high-poverty (HP) city group, derived from piecewise structural equation modeling (pSEM). Socioeconomic factors (Soc_econ in the figure) comprise economic components (poverty rate and GDP per capita) and a social component (access to healthcare). Abbreviations for the variables are provided in Figs. S2 and S13.

Fig. S16. Direct and indirect associations among climate factors, socioeconomic conditions, racial composition, green infrastructure, environmental hazards, and the prevalence of the six health outcomes with the lowest coefficients of determination (R²) in the high-poverty (HP) city group, derived from piecewise structural equation modeling (pSEM). Socioeconomic factors (Soc_econ in the figure) comprise economic components (poverty rate and GDP per capita) and a social component (access to healthcare). Abbreviations for the variables are provided in Figs. S2 and S13.

Fig. S17. Direct and indirect associations among climate factors, socioeconomic conditions, racial composition, green infrastructure, environmental hazards, and the prevalence of the five health outcomes with the highest coefficients of determination (R²) in the moderate-poverty (MP) city group, derived from piecewise structural equation modeling (pSEM). Socioeconomic factors (Soc_econ in the figure) comprise economic components (poverty rate and GDP per capita) and a social component (access to healthcare). Abbreviations for the variables are provided in Figs. S2 and S13.

Fig. S18. Direct and indirect associations among climate factors, socioeconomic conditions, racial composition, green infrastructure, environmental hazards, and the prevalence of the six health outcomes with the lowest coefficients of determination (R²) in the moderate-poverty (MP) city group, derived from piecewise structural equation modeling (pSEM). Socioeconomic factors (Soc_econ in the figure) comprise economic components (poverty rate and GDP per capita) and a social component (access to healthcare). Abbreviations for the variables are provided in Figs. S2 and S13.

Fig. S19. Direct and indirect associations among climate factors, socioeconomic conditions, racial composition, green infrastructure, environmental hazards, and the prevalence of the five health outcomes with the highest coefficients of determination (R²) in the low-poverty (LP) city group, derived from piecewise structural equation modeling (pSEM). Socioeconomic factors (Soc_econ in the figure) comprise economic components (poverty rate and GDP per capita) and a social component (access to healthcare). Abbreviations for the variables are provided in Figs. S2 and S13.

**Fig. S20.** Direct and indirect associations among climate factors, socioeconomic conditions, racial composition, green infrastructure, environmental hazards, and the prevalence of the six health outcomes with the lowest coefficients of determination (R²) in the low-poverty (LP) city group, derived from piecewise structural equation modeling (pSEM). Socioeconomic factors (Soc_econ in the figure) comprise economic components (poverty rate and GDP per capita) and a social component (access to healthcare). Abbreviations for the variables are provided in Figs. S2 and S13.

Fig. S21. Direct and indirect associations among climate factors, socioeconomic conditions, racial composition, green infrastructure, environmental hazards, and the prevalence of the five health outcomes with the highest coefficients of determination (R²) in Group A, one of the city groups identified using Uniform Manifold Approximation and Projection (UMAP), derived from piecewise structural equation modeling (pSEM). Socioeconomic factors (Soc_econ in the figure) comprise economic components (poverty rate and GDP per capita) and a social component (access to healthcare). Abbreviations for the variables are provided in Figs. S2 and S13.

Fig. S22. Direct and indirect associations among climate factors, socioeconomic conditions, racial composition, green infrastructure, environmental hazards, and the prevalence of the remaining six health outcomes with the lowest coefficients of determination (R²) in Group A, one of the city groups identified using Uniform Manifold Approximation and Projection (UMAP), derived from piecewise structural equation modeling (pSEM). Socioeconomic factors (Soc_econ in the figure) comprise economic components (poverty rate and GDP per capita) and a social component (access to healthcare). Abbreviations for the variables are provided in Figs. S2 and S13.

Fig. S23. Direct and indirect associations among climate factors, socioeconomic conditions, racial composition, green infrastructure, environmental hazards, and the prevalence of the five health outcomes with the highest coefficients of determination (R²) in Group B, one of the city groups identified using Uniform Manifold Approximation and Projection (UMAP), derived from piecewise structural equation modeling (pSEM). Socioeconomic factors (Soc_econ in the figure) comprise economic components (poverty rate and GDP per capita) and a social component (access to healthcare). Abbreviations for the variables are provided in Figs. S2 and S13.

Fig. S24. Direct and indirect associations among climate factors, socioeconomic conditions, racial composition, green infrastructure, environmental hazards, and the prevalence of the remaining six health outcomes with the lowest coefficients of determination (R²) in Group B, one of the city groups identified using Uniform Manifold Approximation and Projection (UMAP), derived from piecewise structural equation modeling (pSEM). Socioeconomic factors (Soc_econ in the figure) comprise economic components (poverty rate and GDP per capita) and a social component (access to healthcare). Abbreviations for the variables are provided in Figs. S2 and S13.

Fig. S25. Direct and indirect associations among climate factors, socioeconomic conditions, racial composition, green infrastructure, environmental hazards, and the prevalence of the five health outcomes with the highest coefficients of determination (R²) in Group C, one of the city groups identified using Uniform Manifold Approximation and Projection (UMAP), derived from piecewise structural equation modeling (pSEM). Socioeconomic factors (Soc_econ in the figure) comprise economic components (poverty rate and GDP per capita) and a social component (access to healthcare). Abbreviations for the variables are provided in Figs. S2 and S13.

**Fig. S26.** Direct and indirect associations among climate factors, socioeconomic conditions, racial composition, green infrastructure, environmental hazards, and the prevalence of the remaining six health outcomes with the lowest coefficients of determination (R²) in Group C, one of the city groups identified using Uniform Manifold Approximation and Projection (UMAP), derived from piecewise structural equation modeling (pSEM). Socioeconomic factors (Soc_econ in the figure) comprise economic components (poverty rate and GDP per capita) and a social component (access to healthcare). Abbreviations for the variables are provided in Figs. S2 and S13.
